# Non-invasive neurostimulation techniques for the treatment of stimulant use disorders

**DOI:** 10.3389/fpsyt.2026.1755441

**Published:** 2026-02-04

**Authors:** Jordan Hendy, Hannah Bereznicki, Robert M. Lundin

**Affiliations:** 1 School of Rural Health, Monash University, Mildura, VIC, Australia; 2 School of Psychology, Deakin University, Geelong, VIC, Australia; 3 Alcohol and Other Drugs Integrated Treatment Team, Area Mental Health and Wellbeing Services, Mildura Base Public Hospital, Mildura, VIC, Australia; 4 Academic Regional and Rural Addictions Network (ARRAN), Mildura, VIC, Australia

**Keywords:** addictive disorders, cravings, neurostimulation, stimulant use disorder, substance use disorders, transcranial direct current stimulation, transcranial magnetic stimulation

## Abstract

Addictive disorders remain important contributors to the overall burden of disease, and although many have established treatments, stimulant use disorders (StUDs) still lack effective management options. Neurostimulation techniques, such as Transcranial Magnetic Stimulation and Transcranial Direct Current Stimulation, have attracted addiction medicine researchers and clinicians, with many studies showing promise in reducing cravings and improving other clinical outcomes in participants, as well as modulating relevant brain areas. As a result, research output in this area is increasing rapidly. This narrative review aims to assess currently available research data on non-invasive neurostimulation techniques in patients with StUDs to inform future research requirements and clinical applications. This review was conducted using a comprehensive search strategy across PubMed, OVID Medline and PsycINFO databases, using terms including “stimulant use disorder*”, “transcranial magnetic stimulation” and “craving*”. The initial search was intentionally broad to effectively assess the breadth of literature on neurostimulation in addiction disorders generally, resulting in the return of 1317 sources. Search results were uploaded to Covidence and screened for inclusion. Upon narrowing the scope to isolate StUDs, 179 sources were included for full-text review, with 90 included for extraction. The most common outcome measure assessed was craving, with cognition, affective symptoms, sleep, and use patterns other frequently assessed measures. Overall, the results appear promising, with non-invasive neurostimulation variably improving cravings, cognition and affective outcomes in patients with StUDs. However, this review identified some limitations that require further research attention, including small sample sizes, short follow-up periods, protocol heterogeneity, and ill-representative samples.

## 1 Introduction

Substance Use Disorders (SUDs) are a recognised category of mental disorder in the Diagnostic and Statistical Manual of Mental Disorders (DSM-5-TR) and remain a prevalent global challenge. Their diagnostic criteria cover four main categories of symptoms relating to impaired control, physical dependence, social problems and risky use (1). Within Australia, SUDs are the third most prevalent mental disorder, affecting a significant proportion of the adult population (2). SUDs contribute a significant burden to the individuals affected, their families and broader society, with further comorbid mental health conditions commonly observed in this population. According to the Australian Institute of Health and Welfare (AIHW), the cost of addiction in 2021 was an estimated $80.3 billion, with this figure encompassing several areas, including prevention, treatment, crime and productivity, highlighting the importance of effective management of SUDs (3).

Currently, management options for SUDs mainly consist of behavioural therapies such as Cognitive Behavioural Therapy (CBT) and motivational interviewing, and various pharmacotherapies depending on the addictive substance. Efficacious therapies exist for nicotine, opioid, heroin and alcohol addictions. There are fewer established treatment options for methamphetamine, amphetamine and cocaine addictions, with no medication currently approved by the Food and Drug Administration or the Therapeutic Goods Administration (4), leaving clinicians with only behavioural therapies or off-label pharmacotherapies in their toolkit for managing patients with these addictions.

Stimulant Use Disorder (StUDs) encapsulate a subset of addiction disorders pertaining to methamphetamine, amphetamine and cocaine dependency (4). The AIHW estimated a total expenditure of over $5 billion between 2013–2014 on methamphetamine use alone, highlighting its financial and societal burden (3). Furthermore, medically, stimulants can increase the risk of cardiovascular, cerebrovascular, respiratory, infectious and kidney disease, as well as cause damaging effects to psychological wellbeing through resulting psychosis, depression and/or anxiety (5).

Neurostimulation is a broad term encompassing the expanding repertoire of neuromodulation technologies, with some of the most commonly used methods being Transcranial Magnetic Stimulation (TMS) and Transcranial Direct Current Stimulation (tDCS) (6). These technologies have attracted much interest in the addiction medicine space since the early proposition of their utility for SUDs back in the early 2000s. A collection of literature has been published on its use in the treatment of various SUDs, including nicotine/smoking cessation, alcohol use disorder, opioid addiction and, more scarcely, StUDs, with many studies revealing promising results (7, 8).

The mechanism by which neurostimulation alters addiction pathways and behaviours is not yet fully understood, however, proposed theories include modification of neuronal activity, pathways and functional connectivity between various brain circuits (9). Further research is required to fully establish how neurostimulation has produced the clinical outcomes observed in previous studies to optimise benefit and treatment trajectories further.

TMS was first applied clinically in the late 1990s, operating according to Faraday’s laws (9, 10). The technology utilises a coil applied to the surface of the scalp, which transmits magnetic pulses in a way that induces electrical currents in the targeted brain area. Over time, the technique has expanded to include other variations such as repetitive transcranial magnetic stimulation (rTMS), deep transcranial magnetic stimulation (dTMS) and intermittent theta burst stimulation (iTBS). iTBS has been shown to be equally efficacious as traditional TMS but can reduce the average standard 20-minute treatment session to three minutes (11). Alternatively, tDCS uses electrical currents to modulate neuronal messaging rather than magnetic currents and involves electrode placement on the scalp (12).

The three broad regions of the brain most closely associated with addiction pathophysiology are the basal ganglia, the extended amygdala, and the prefrontal cortex (PFC) (13). The PFC has multiple important subregions, including the Dorsolateral Prefrontal Cortex (DLPFC) and the Ventromedial Prefrontal Cortex (vmPFC) (14), with its overarching role of executive functioning making it the main operator in the preoccupation/anticipation stage of addiction (13). This region is responsible for cravings, and its dysfunction in addiction explains why affected individuals find it difficult to control urges to engage in substance use, especially when exposed to drug-related cues (13). These same individuals often show deficits in the areas of the PFC that help regulate stress and emotional systems in the brain, further reducing their ability to oppose cravings (13). Important networks also span across the PFC, including the Executive Control Network (ECN), the Default Mode Network (DMN), and the salience Ventral Attention Network (VAN), all of which influence the processing of stimuli (15).

The primary region that has been targeted by neurostimulation in SUDs is the DLPFC, in both the left and right hemispheres of the brain. This region of the brain is specifically involved in reward, motivation and decision-making, as well as cognitive control and inhibition, making it particularly relevant in drug cravings and use (16). The most common EEG signal investigated in neurostimulation and addiction studies is P3, which has been associated with levels of attentional bias towards cues (17).

A recent article published in Frontier Psychiatry provides an overview of how neuromodulation can form part of the treatment toolkit for opioid and StUDs, most relevant to this review, they again highlight the absence of an FDA-approved treatment for StUD (18). Whilst providing a useful overview of the current space, the aforementioned article only included two studies which investigated non-invasive neurostimulation therapies in participants with StUD. As such, the current review was developed to investigate the wider body of emerging literature to better elucidate non-invasive neurostimulation’s potential for the management of StUDs.

This review aimed to assess what is currently known regarding the use of non-invasive neurostimulation techniques in the treatment of StUDs. We sought to review: (1) how non-invasive neurostimulation impacts clinical outcomes in patients with StUDs, (2) the modalities being assessed in the current evidence base, and (3) how these modalities compare to one another.

Secondary areas of interest included which target site(s) are frequently assessed, whether variations in these are related to clinical outcomes, and whether neuroimaging has better informed our understanding of the mechanism of such treatments.

## 2 Methods

### 2.1 Search strategy

A narrative review format was chosen for this review to allow for a broad assessment of the research space and analysis of available literature. In the initial stages of the review, the research question was intentionally broad to include sources on neurostimulation in SUD treatment more broadly, to ascertain the currently available literature on what was anticipated to be a sparse area of research. This was achieved using an array of search terms such as “substance use disorder*”, “cocaine use disorder”, “methamphetamine use disorder”, “stimulant use disorder”, “transcranial magnetic stimulation”, “transcranial direct current stimulation”, “craving*”, “abstinen*”, “withdrawal symptoms”. A comprehensive summary of all terms used is shown in **Appendix A**. The search was conducted across three databases, PubMed, OVID Medline and PsycINFO, which were chosen due to their applicability to our field of research. The publication date range was set to 2003-present, as 2003 marked the initial proposal of neurostimulation for addiction management. All sources were then uploaded into Covidence for screening purposes where screening was conducted by a single reviewer (JH). Whilst a single reviewer (JH) completed the screening process, frequent discussions took place between this reviewer and another author (RML) when any uncertainty regarding a study’s inclusion or exclusion arose to ensure all relevant literature was assessed as part of this review.

### 2.2 Eligibility criteria

After reviewing the broader research output, we adjusted our inclusion criteria to limit our review to resources exploring non-invasive neurostimulation modalities in managing StUDs, given the limited current management options compared to other SUDs. Sources were included if they assessed non-invasive neurostimulation modalities in the management of StUDs in human participants, were a peer-reviewed systematic review, literature review, randomized-controlled trial, observational study, or case report, and assessed clinical outcomes relevant to StUDs.

Given the clinical interest of this review in noninvasive neurostimulation modalities for StUDs, sources were excluded if their primary focus was on an addictive substance other than cocaine or methamphetamines, were based upon animal studies, explored invasive neurostimulation techniques such as Deep Brain Stimulation or vagus nerve stimulation. Book chapters, letters to the editor, and commentaries were excluded as they were deemed not rigorous enough.

The same single reviewer conducted the screening and extracted and analysed evidence from included studies with assistance from a co-author (HB) to assess trends and overall findings. No quality assessments were performed so as not to hinder scoping assessment in order to gain a broader overview of an evolving field where there is limited consensus on standardized protocols, targets and impact. As such, this allowed the review to better demonstrate how the field has been evolving over the relatively short period of time. Therefore, no studies were excluded due to low quality.

## 3 Results

### 3.1 Study characteristics

**Figure 1:**
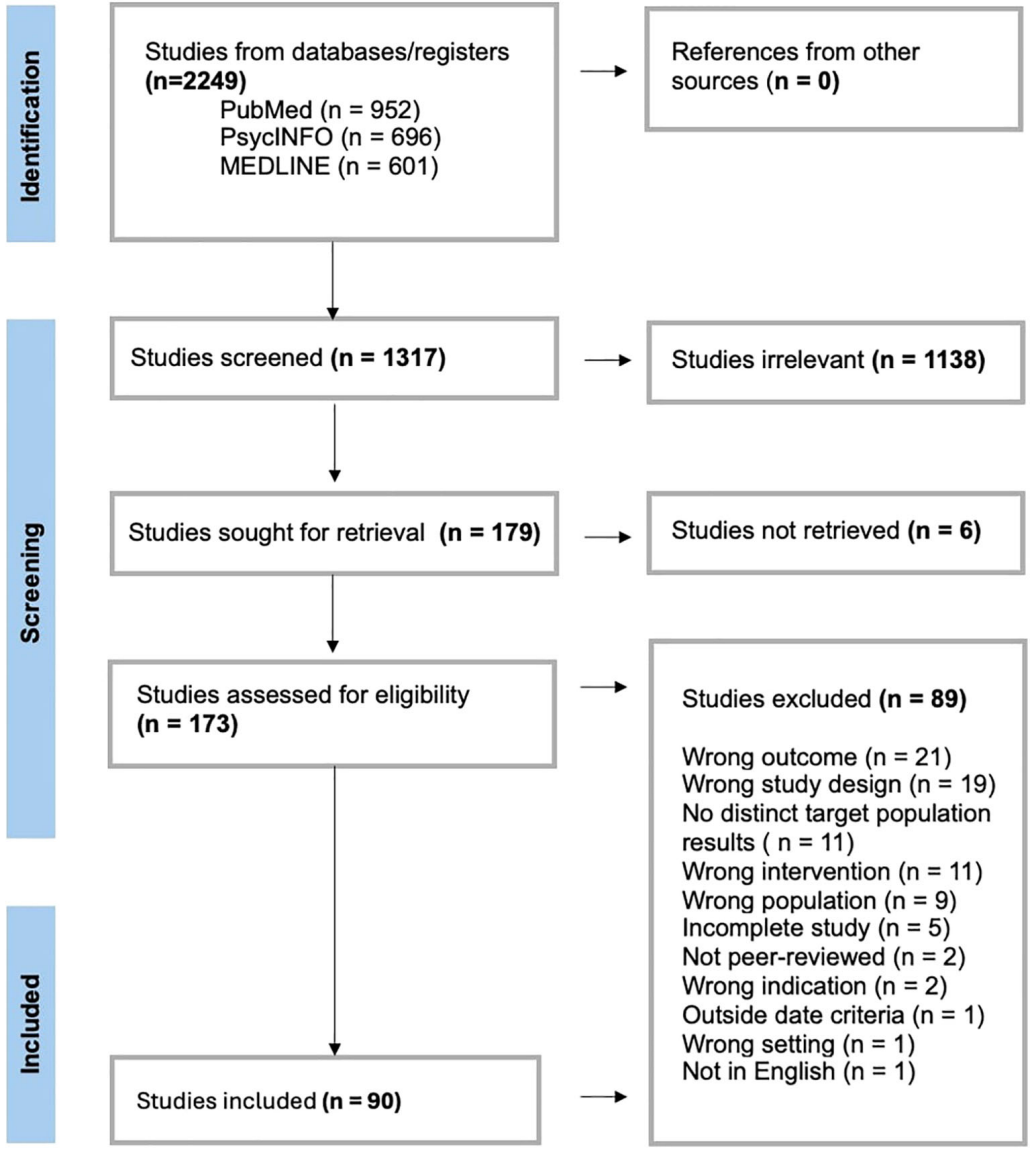
PRISMA flowchart detailing screening and inclusion/exclusion process for returned search results using covidence.

The initial search returned 1,317 studies after being processed through Covidence, and manual screening removed duplicates. Using the above inclusion and exclusion criteria, 179 studies were deemed suitable for inclusion at the title and abstract screening phase, and 90 were subsequently included for extraction, as shown in the PRISMA flowchart below (**Figure 1**).

Across the 90 studies, 57 were primary studies with a total of 3,319 participants, and 33 were reviews. Primary studies are summarised in **Tables 1**–**4**, with a more extensive presentation of findings recorded in **Supplementary Tables 1–4**. Review studies are summarised in **Supplementary Tables 1–4**.

**Table 1.1:** Studies reporting clinical outcomes with a sham condition.

Author, Year	n	Intervention^comparator^	Outcomes	Findings
Studies with clinical outcomes only				
Fayaz Feyzi et al., 2022 (21)	60 M	tDCS + Matrix Model psychotherapy^SN; N^	Clinical^a,d,e,g^	Active tDCS group showed a significant reduction in craving score compared to other groups. Cognition significantly improved in the active tDCS group compared to baseline, although no significant difference was found between groups. Non-significant reduction in relapse rates in active tDCS group and the other two gorups. Nil serious adverse events, main reported side effect was tingling.
Jiang et al., 2022 (70)	45 M	tDCS^S^	Clinical^f^	Increased impulsivity in the active tDCS group from baseline. The same was not seen in sham.
Martinotti et al., 2022 (22)	80 C	Initial rTMS+ maintenance rTMS^S^	Clinical^a,b,e,g,h^	Active and sham showed a significant reduction in cravings and withdrawal symptoms. Active and sham had non-significantly lower incidence of positive drug urine tests. Active rTMS group had a significantly greater reduction in depressive symptoms compared to sham, and this reduction was even greater in those who underwent > 40 sessions and/or took psychotropics at the same time. Nil serious adverse events.
Wang et al., 2022 (23)	64 M	10-Hz rTMS^S^	Clinical^a,d,g^	Significantly lower cravings, and significantly improved block 4 and 5 on the IGT in active rTMS group compared to pre-treatment and to sham. Well tolerated.
Alizadehgoradel et al., 2021 (24)	80 M	tDCS^AN;N;S^	Clinical^a,d,g^	Both immediately after intervention at 1-month follow-up, performance in most EF tasks were significantly better in the combination group compared to baseline and other groups. Significant decrease in cravings in all active intervention groups until 1-month follow-up. Nil serious adverse events.
Garza-Villarreal et al., 2021 (25)	44 C	rTMS + standard treatment followed by maintenance rTMS + standard treatment^SN^	Clinical^a,b,c,e,f^	Statistically significant reduction in craving and impulsivity in the active group. Anxiety, depression and sleep quality improved significantly in both groups. Clinical effects were maintained for 3 months but were lost by the 6-month endpoint. No significant change in urine drug test results.
Gaudreault et al., 2021 (26)	17 C	tDCS^S^	Clinical^a,b,c,e,f,g,i,j^	Non-significant decrease in cravings greater in active tDCS vs sham; expected to reach significance if n> 15 per group. Sleepiness and readiness to change showed significant, and non-significant change respectively in the active tDCS group. Quality of life and impulsivity improved in both groups. No significant changes in depression or anxiety. Nil serious adverse events, main reported side effect was mild tingling.
Lolli et al., 2021 (27)	62 C	rTMS^S^	Clinical^a,b,e,f,g^	No significant difference in positive urine drug tests. Self-reported days of use improved in both groups but was significantly lower in active vs sham. Impulsivity and depression scales improved in both groups. Cue-induced craving was significantly decreased in the active group, current craving was significantly reduced in the sham group. Mild side effects only.
Alizadehgoradel et al., 2020 (28)	39 M	tDCS^S^	Clinical^a,d,g^	Significantly better performance on all EF tasks following active tDCS when compared to their own baseline and to the sham group, at both immediately after the intervention and at 1 month follow-up. Active tDCS group had a significant decrease in cravings. Well tolerated.
Su et al., 2020 (29)	126 M	iTBS^S^	Clinical^a,c,d,e^	The active iTBS group showed significant improvements in cravings,and sleep compared to sham, and significant improvements to various cognitive functions compared to either sham or baseline. At 3 months 9.6% vs 3.1% relapsed in sham vs active iTBS.
Verveer et al., 2020 (30)	59 C	tDCS^S^	Clinical^a,d,e^	Significant decrease in cravings across both active and sham. No effect on cognition. Significant reduction in use in the active group participants who used crack-cocaine specifically (not powdered) compared to sham.
Yuan et al., 2020 (31)	106 M	rTMS^S^	Clinical^a,f^	Accuracy in the 2-choice oddball task was significantly increased in the active rTMS group compared to baseline, with a corresponding significant decrease in craving.
Anaraki et al., 2019 (32)	30 M	tDCS^S^	Clinical^a,b^	Cue-induced craving was significantly decreased in the active tDCS group. No change in immediate craving. Insignificant improvement in affect.
Klauss et al., 2018 (33)	35 C	tDCS^S^	Clinical^a,e,g^	Craving scores significantly decreased in both groups. Relapse rates were similar amongst both groups (41.7% sham vs 41.2% active). Scalp tinging experienced by 63.3% of participants with no difference between active and sham. Nil serious adverse events.
Su et al., 2017 (34)	30 M	rTMS^StDCS^	Clinical^a,b,c,d^	Craving was significantly reduced, and cognition improved in the active rTMS group compared to sham. Depression significantly decreased in both groups. No significant change to anxiety or sleep.
Bolloni et al., 2016 (66)	10 C	rTMS + weekly psychological support^SN^	Clinical^e^	Significant reduction in cocaine amount detected in hair samples up to 3 months post rTMS compared to baseline.
Batista et al., 2015 (35)	36 C	tDCS^S^	Clinical^a,b,g,j^	Craving was significantly decreased, and QOL significantly increased in the active group compared to sham. Significant decrease in anxiety and nonsignificant difference in depression in the active group compared to sham. Active group has significantly improved depression compared to baseline. Mild side effects only. Scalp tingling in 72.2% active; 73.7% sham.
Shahbabaie et al., 2014 (36)	31 M	tDCS^S^	Clinical^a,g^	Significant reduction in at rest craving after 10 minutes of active tDCS compared to sham. Cue-induced craving increased in active tDCS vs sham. Well tolerated by all participants. Main side effects were scalp tingling and drowsiness.
Li et al., 2013 (48)	18 M	rTMS^S^	Clinical^a,g^	Increased craving after active rTMS compared to sham. Mild transient side effects only.
Studies with target site focus that also report clinical outcomes				
Hou et al., 2025 (37)	60 M	HD-tDCS^S^	Clinical^a,g^	Significant decreases in craving post active HD-tDCS up to 1-month follow-up compared to baseline and sham, including a significant reduction in heart rate. Well tolerated.
Chen et al., 2020 (38)	74 M	Group A: iTBS^S^ Group B: cTBS^S^ Group C: iTBS + cTBS^S^	Clinical^a,b,c,d,h^	Non-significant improvements in depressive symptoms and sleep quality. Significant improvements in withdrawal symptoms in Group C vs sham. Active TBS significantly reduced craving compared to sham. No effect was seen on cognition. Anxiety improved the most in Group C (significantly).
Shahbabaie et al., 2018 (68)	90 M	tDCS^S^	Clinical^b,d^	Active tDCS significantly reduced attentional bias towards drug cues compared to sham. Non-significant changes to mood.
Studies with an MRI focus that also report clinical outcomes				
McCalley et al., 2024 (20)	33 C	cTBS + behavioural counselling^SN^	Clinical^a,b,e,f,g^	Abstinence was greater in the intervention group compared to control, although not statistically significant. No change to craving, anxiety, depression or impulsivity. Well tolerated, nil major side effects.
Rasgado-Toledo et al., 2024 (39)	50 C	rTMS^S^	Clinical^a^	Non-significant changes in craving from baseline and post rTMS, however significant changes were predicted by predictive modelling based on baseline white matter microstructure.
Ekhtiari et al., 2022 (40)	60 M	tDCS^S^	Clinical^a,g^	No changes to cravings. Mild side effects, most commonly sleepiness and tingling.
Soleimani et al., 2022 (15)	15 M	tDCS^S^	Clinical^a,b,g^	Cravings significantly decreased in the active rTMS group compared to sham. Non-significant changes to affect. No serious adverse events.
Su et al., 2020 (42)	50 M	rTMS^S^	Clinical^a,d^	Significant improvement in cognitive function. Cravings were significantly decreased in active but not in sham.
Su et al., 2020 (41)	60 M	iTBS^S^	Clinical^a^	Significant reduction in craving compared to sham.
Kearney-Ramos et al., 2019 (43)	19 C	cTBS^S^	Clinical^a^	Insignificant decrease in craving
Shahbabaie et al., 2018 (44)	15 M	tDCS^S^	Clinical^a,g^	Craving decreased significantly in active tDCS compared to sham. Mild side-effects only, well tolerated.
Studies with an EEG focus that also report clinical outcomes				
Li et al., 2024 (45)	51 M	rTMS^S;C^	Clinical^a^	Significant reductions in craving level in active group from baseline.
Chen et al., 2023 (69)	30 M	tDCS^S;C^	Clinical^b,d^	Insignificant effect was noted on behavioural performance. tDCS significantly reduced disengagement scores. Significantly improved mood regulation in active compared to sham. No significant differences in affective symptoms between groups.
Khajehpour et al., 2022 (17)	42 M	tDCS^S^	Clinical^a,b^	Significant reduction in craving from baseline. Significant improvement to negative affect in both groups.
Wen et al., 2022 (46)	15 M	N+iTBS^S+N^	Clinical^a^	Significant reduction in craving in the active iTBS group compared to sham.
Chen et al., 2021 (47)	49 M	iTBS^S^	Clinical^a,d^	Significantly reduced error rate in the active group compared to sham. No significant differences in reaction time between groups. Significant reduction to cravings in active vs sham.

**Table 1.1** presents the clinically relevant outcomes and findings of all included sham-controlled studies. The sham condition and methodology of these studies place greater weighting on their findings as opposed to the remaining studies, which were methodologically weaker. Cravings were the most consistently significantly improved outcome across studies. Other outcomes including cognition, affective symptoms, sleep and use patterns were more variably affected by neurostimulation.

Sample (n) key: C, Cocaine Use Disorder, M, Methamphetamine Use Disorder.

Clinical outcomes: ^a^cravings, ^b^affective symptoms, ^c^sleep, ^d^cognition, ^e^substance use, ^f^Impulsivity, ^g^tolerability, ^h^withdrawal symptoms, ^i^readiness to change, ^j^Quality of Life (QOL).

Comparators: ^C^healthy control, ^S^sham neurostimulation, ^A^active eurostimulation ^N^non-neurostimulation treatment.

**Table 1.2:** Studies reporting clinical outcomes without a sham condition.

Author, Year	n	Intervention^comparator^	Outcomes	Findings
Studies reporting clinical outcomes only				
Cardullo et al., 2024 (49)	126 C	rTMS^C^	Clinical^a,b,c,d,e^	Significant improvement in craving, anxiety, depression and sleep in CUD patients from their baseline compared to healthy controls. Temporal variability decreased following rTMS.
Liu et al., 2024 (50)	89 M	rTMS^C^	Clinical^a,d^	Significant decrease in craving score and non-significantly improved decision making after rTMS.
Liu et al., 2022 (60)	58 M	HF-rTMS^C^	Clinical^a,d^	Significantly decreased craving and reaction time after HF-rTMS. No significant change in response inhibition.
Sanna et al., 2022 (67)	89 C	iTBS^Z^	Clinical^e,g^	At 12 months, 69.7% were abstinent, 30.3% relapsed. During acute phase of iTBS, majority of participants had consistently negative drug urine tests. Maintenance therapy significantly reduced drop out rates, however did not significantly impact relapse rates compared to non-maintenance. Well tolerated, nil serious adverse events.
Cardullo et al., 2021 (51)	230 C	HF-rTMS^Z^	Clinical^a,b,c,e^	All participants showed significant improvement across all outcomes. No significant differences between participants with or without ADHD.
Gómez Pérez et al., 2020 (52)	87 C	rTMS^Z^	Clinical^a,b,c,e,g,h^	All outcomes significantly improved in the rTMS group. Degree of sleep improvement was correlated with number of rTMS sessions. No significant changes were observed in the waitlist group. No serious adverse events.
Liu et al., 2019 (53)	90 M	rTMS + Routine addiction Rehabilitation^N^	Clinical^a,g^	Significant reduction in craving in the rTMS group compared to controls at 1 month follow-up. However both groups showed significant reduction from their respective baselines. Well tolerated
Pettorruso et al., 2019 (54)	20 C	Initial rTMS + maintenance rTMS^Z^	Clinical^a,b,c,g,h^	Significant reduction in psychopathological distress, withdrawal symptom, urine drug tests, reported use and cravings. Significant reduction in overall psychopathological burden and depression. non-significant improvements in sleep and suicidal ideations compared to badeline. Nil serious side effects.
Steele et al., 2019 (65)	19 C	iTBS^Z^	Clinical^a,e,g^	9 patients reduced cocaine consumption. A reduction in use of other substances such as THC and alcohol was also observed. Cravings insignificantly decreased during iTBS treatment period. Well tolerated. Main side effect was occasional headaches. 1 transient neurological event of unclear aeiteology and one cocaine-induced psychosis two weeks after iTBS discontinuation.
Rapinesi et al., 2016 (55)	7 C	dTMS + prior drug treatment^Z^	Clinical^a,g^	Significant reduction in craving up to 2 months post end of treatment. Tolerated by all participants. Nil serious side effects.
Shariatirad et al., 2016 (56)	1 M	tDCS^Z^ +/- Booster tDCS sessions	Clinical^a,b,d,e,h^	Considerable improvement in cravings, cognition and depressive symptoms. 3 lapses reported across the 6 months with 4 booster sessions required on days 67, 70, 72, 88.
Terraneo et al., 2016 (57)	32 C	Stage 1: rTMS^N^ Stage 2: rTMS (both groups from stage 1)	Clinical^a,b,e,g^	Stage 1: Significantly more negative urine tests, and reduced craving in the rTMS group compared to the control. Stage 2: Significant improvement in almost all outcomes following rTMS. Insignificant improvement in depression in both groups. Mild side effects only, mostly scalp discomfort.
Studies with a target site focus that also report clinical outcomes				
Rezvanian et al., 2022 (16)	15 M	tDCS^P^	Clinical^a,d^	Significant increase in cognitive inhibition and presentation error. Non-significant decrease in craving.
Liu et al., 2017 (58)	50 M	rTMS^P^	Clinical^a^	rTMS significantly reduced cue-induced craving.
Camprodon et al., 2007 (59)	6 C	rTMS^Z^	Clinical^a,b^	rTMS significantly reduced craving, however this disappeared after 4 hours. Significant reduction in anxiety and improvements in mood.
Studies with a neurostimulation modality focus that also report clinical outcomes				
Liu et al., 2022 (11)	20 M	iTBS^rTMS^	Clinical^a,b,h^	Both modalities significantly improved cravings and withdrawal symptoms. rTMS significantly improved affective symptoms, iTBS did not, however no overall difference was found between groups.
Zhao et al., 2020 (61)	83 M	iTBS^cTBS^	Clinical^a,b,c,f,g^	Non-significant reduction in craving with both modalities. Significant improvement in depression and sleep. iTBS only in anxiety. Mild adverse only, incidence higher in iTBS compared to cTBS. Insignificant effects on impulsivity
Sanna et al., 2019 (62)	47 C	iTBS^rTMS^	Clinical^a,e,g^	Significant reduction in cravings and use in both groups. Mild side effects only. Most common side effects were sleepiness and headache.
Studies with a neuroimaging (MRI +/- EEG) focus that also report clinical outcomes				
Zhang et al., 2025 (63)	227 C	rTMS^C^	Clinical^a^	rTMS non-significantly reduced cravings.
Nakamura-Palacio et al., 2016 (64)	14 C	tDCS^Z^	Clinical^a^	Non-significant reduction in craving.

**Table 1.2** presents the clinically relevant outcomes and findings reported by included studies that did not employ a sham condition. Their respective findings should be interpreted and weighed accordingly, taking into account the limitation of an absent sham-controlled group. Cravings were the most consistently improved outcomes across studies.

Sample (n) key: C, Cocaine Use Disorder, M, Methamphetamine Use Disorder.

Clinical outcomes: ^a^cravings, ^b^affective symptoms, ^c^sleep, ^d^cognition, ^e^substance use, ^f^Impulsivity, ^g^tolerability, ^h^withdrawal symptoms.

Comparators: ^C^healthy control, ^N^non-neurostimulation treatment, ^Z^no treatment, ^P^varied protocol parameters.

**Table 2:** Findings of studies comparing neurostimulation modalities.

Author, Year	n	Intervention	Outcomes	Findings
Liu et al., 2022 (11)	20 M	iTBS^rTMS^	Clinical^a,b,h^	iTBS and 10 Hz rTMS resulted in similar significant improvements in craving and withdrawal symptoms. rTMS significantly improved affective symptoms. No significant differences between iTBS vs rTMS.
Zhao et al., 2020 (61)	83 M	iTBS over left DLPFC^cTBS^ Vs cTBS over right DLPFC^cTBS left^	Clinical^a,b,c,f,g^	iTBS of the left DLPFC and cTBS of the right DLPFC significantly reduced craving. No main effects were seen in impulsivity. All groups showed significant decreases in depressive symptoms, only the iTBS had a decrease in anxiety. Both iTBS and L cTBS significantly improved sleep. Adverse effects were higher in the left iTBS group.
Sanna et al., 2019 (62)	47 C	iTBS^rTMS^	Clinical^a,e^	Virtually identical significant effects on outcomes observed between iTBS and rTMS, with similar side effects and dropout rates. Significant reduction in cravings and use post either treatment.

**Table 2** presents outcomes and findings reported by included studies that compared various neurostimulation modalities to one another. None of the above studies employed a sham group. No study identified a clearly superior modality.

Sample (n) key: C, Cocaine Use Disorder, M, Methamphetamine Use Disorder.

Clinical Outcomes: ^a^cravings, ^b^affective symptoms, ^c^sleep, ^e^substance use, ^f^impulsivity, ^g^tolerability, ^h^withdrawal symptoms.

**Table 3:** Findings of studies comparing target sites.

Author, Year	n	Target site(s)	Intervention	Outcomes	Findings
Hou et al., 2025 (37)	60 M	Right vs Left DLPFC	HD-tDCS^S^	Clinical^a^	No difference between left and right DLPFC. Significant decreases in craving post active HD-tDCS up to 1-month follow-up.
Zhao et al., 2023 (100)	337 C	Personalised montage over left DLPFC	rTMS^S^	Treatment response with diagnostic FC signature	Applying the functional connectivity signature to rTMS therapy was found to be predictive for treatment response.
Rezvanian et al., 2022 (16)	15 M	6 varied electrode montages (details in **Supplementary Table 3**)	tDCS^P^	Clinical^a,d^	Significant increase in cognitive inhibition, protocol 2 superior. Significant improvement for presentation error, protocol 4 superior. No significant change to craving.
Chen et al., 2020 (38)	74 M	Group A: Left DLPFC vs Group B: Left vmPFC vs Group C: A+B	Group A: iTBS^S^ Group B: cTBS^S^ Group C: iTBS + cTBS^S^	Clinical^a,b,c,d,h^	Significant improvement in withdrawal and anxiety symptoms. Group C > Group A. Non-significant improvements in sleep and depressive symptoms compared to sham TBS significantly reduced craving compared to sham regardless of site. No effect on cognition.
Shahbabaie et al., 2018 (68)	90 M	5 varied electrode montages (details in **Supplementary Table 3**)	tDCS^S^	Clinical^b,d^	Left DLPFC/right shoulder and left DLPFC/right DLPFC reduced attentional bias towards drug cues compared to sham. No significant differences in affective symptoms between groups.
Liu et al., 2017 (58)	50 M	Left P3 Vs Left DLPFC Vs Right DLPFC	rTMS - low and high frequency^P^	Clinical^a^	rTMS significantly reduced craving across all conditions
Camprodon et al., 2007 (59)	6 C	Right vs left DLPFC	rTMS^Z^	Clinical^a,b^	Right, but not left, DLPFC stimulation significantly reduced craving. This disappeared after 4 hours. Significant improvement in affective symptoms.

**Table 3** presents outcomes and findings reported by included studies that compared different neurostimulation target sites to one another. Four of the above studies employed a sham condition) (37, 38, 68, 100), whilst the remaining three did not (16, 58, 59). Findings for site to site comparisions were varied.

Sample (n) key: C, Cocaine Use Disorder, M, Methamphetamine Use Disorder.

Clinical outcomes: ^a^cravings, ^b^affective symptoms, ^c^sleep, ^d^cognition, ^h^withdrawal symptoms.

Comparators: ^S^sham neurostimulation, ^Z^no treatment, ^P^varied protocol parameters.

**Table 4.1:** Findings of studies with an MRI neuroimaging focus.

Author, Year	n	Intervention^Comparator^	MRI type	Imaging outcomes	Findings
Zhang et al., 2025 (63)	227 C	rTMS^C^	fMRI	fMRI changes	At baseline CUD patients showed elevated gradient values in the ventral striatum. rTMS significantly normalised these values and this was significantly correlated with decreases in craving.
Zhao et al., 2023 (100)	337 C	rTMS with Personalised montage^S^	MRI	Treatment response with diagnostic FC signature	Functional connectivity signature was found to be predictive for treatment response.
McCalley et al., 2024 (20)	33 C	cTBS + behavioural counselling^SN^	fMRI	Brain activity in response to cues	Reduced activity in MPFC*, insula and anterior cingulate in active cTBS group. No change to craving.
Rasgado-Toledo et al., 2024 (39)	50 C	rTMS^S^	T1-WI and HARDI**	MRI changes to white matter (WM) microstructure in the frontostriatal circuits	rTMS resulted in significant increases in neurite density connection and reduced orientation dispersion between various brain regions. No correlated changes in craving or impulsivity.
Ekhtiari et al., 2022 (40)	60 M	tDCS^S^	fMRI	Brain activity changes	Decreased brain activity in response to drug cues after sham. Significant increase in brain activity in response to drug cues and stronger connections within the frontoparietal network after active tDCS No significant changes to cravings.
Soleimani et al., 2022 (15)	15 M	tDCS^S^	Structural + fMRI	MRI changes to 3 brain areas, the ECN, DMN, VAN***	tDCS increased activity and communication between the ECN and VAN, whilst it decreased the activity and communication between the DMN and VAN. Cravings decreased significantly in the active tDCS group.
Garza-Villarreal et al., 2021 (25)	44 C	rTMS + standard treatment^Z^ Maintenance rTMS for up to 6 months	fMRI	Brain changes	Increased connectivity between left DLPFC and vmPFC, and between vmPFC and right angular gyrus following rTMS. These effects remained until 3 months and were gone by 6 months. Craving significantly reduced.
Su et al., 2020 (42)	50 M	rTMS^S^	H-MRS****	GABA + Resonance of glutamate + glutamine (Glx) relative to n-acetyl-aspartate (NAA)	Significant reductions in GABA/NAA in the active rTMS group. Significant reduction in Glx and NAA in sham not active. Significant association between reductions in GABA and improvement in cognitive function. Cravings were significantly decreased in active rTMS.
Su et al., 2020 (41)	60 M	iTBS^SrTMS^	fMRI	Connectivity of brain areas	Increased connectivity between left DLPFC and inferior parietal lobule in iTBS, correlating with a significant reduction in craving. Decreased connectivity between insula and inferior parietal lobule, medial temporal lobe and precuneus in those in iTBS.
Kearney-Ramos et al., 2019 (43)	19 C	cTBS^S^	fMRI	Brain changes during cue exposure	Decrease in striatum activity following active cTBS when exposed to cues. No overall significant changes to craving.
Shahbabaie et al., 2018 (44)	15 M	tDCS^S^	fMRI	fMRI focused on ECN, DMN and salience network (SN)	Craving decreased significantly in active tDCS compared to sham and this correlated with the changes observed in brain networks after active tDCS.
Nakamura-Palacios et al., 2016 (64)	14 C	tDCS^Z^	EEG MRI	Brain changes	Strengthened connections between vmPFC and nucleus accumbens after tDCS, and this was related to a reduction in craving.
Conti et al., 2014 (101)	13 C	tDCS^S^	EEG	Brain activity – P3	P3 current density in DLPFC increased during neutral cues and decreases during cocaine-cues after a single session. The opposite was seen in sham. After repetitive tDCS, current density increased when exposed to cocaine-cues.

**Table 4.1** presents MRI neuroimaging-related outcomes and findings reported by included studies. Ten of the above studies employed a sham condition (15, 20, 39–44, 100) whilst the remaining two did not (63, 64). Neurostimulation was found to alter brain activity or connectivity in some way across all studies.

*Medial Prefrontal Cortex.

**T1- weighted and high angular resolution diffusion-weighted imaging.

***ECN, Executive Control Network, DMN, Default Mode Network, VAN, Ventral Attention Network.

**** Proton magnetic resonance spectroscopy.

Sample (n) key: C, Cocaine Use Disorder, M, Methamphetamine Use Disorder.

Comparator: ^C^Healthy controls, ^S^sham neurostimulation, ^N^non-neurostimulation treatment, ^Z^no treatment.

**Table 4.2:** Studies with a neuroimaging focus – EEG.

Author, Year	n	Intervention^comparator^	Imaging outcomes	Findings
Li et al., 2024 (45)	51 M	rTMS^S;C^	Changes in microstates	2 of the 4 microstates showed significant improvements after rTMS and these were found to be significantly correlated with reductions in craving level.
Chen et al., 2023 (69)	30 M	tDCS^S;C^	P300 amplitudes	tDCS significantly increased amplitude of P300 to neutral cues, trending towards healthy controls. P300 amplitude insignificantly increased to drug-related cues. No significant effect on behavioural performance, however tDCS significantly reduced disengagement scores.
Khajehpour et al., 2022 (17)	42 M	tDCS^S^	P3 and Late Positive Potential (LPP)	P3 amplitude significantly decreased after active tDCS in response to drug-related cues, whilst it increased in the sham group. No significant change to LPP. Significant changes to craving.
Wen et al., 2022 (46)	15 M	iTBS^S^	Theta: Beta ratio	Significant reduction in Theta:beta ratio in active iTBS group. Significant reduction in craving in the active iTBS group.
Chen et al., 2021 (47)	49 M	iTBS^S^	Addiction Stroop Task whilst EEG monitoring	Reduced error rate in active group compared to sham. Active group had insignificantly stronger P3 amplitudes (connected to a faster response on task) and a reduction in beta-wave activity in response to drug-cues compared to the sham group. Cravings correlated to changes in N1 amplitude on EEG.
Conti et al., 2014 (101)	13 C	tDCS^S^	Brain activity during neutral or crack-related cues	After a single session of active tDCS, P3 current density increased in the left DLPFC during neutral cues and decreased during crack-related cues. The opposite was seen in sham. Following repeated tDCS sessions, P3 was increased in left DLPFC as well as other important brain areas in response to crack-related cues.
Nakamura-Palacios et al., 2016 (64)	14 C	tDCS^Z^	Brain changes	Brain connections between vmPFC and nucleus accumbens became stronger after real tDCS and this was related to a reduction in craving.

**Table 4.1** presents EEG neuroimaging-related outcomes and findings reported by included studies. Six of the above studies employed a sham condition) (17, 45–47, 69, 101), whilst the remaining study did not (64). All studies reported some change in EEG results post-neurostimulation.

Comparator: ^C^Healthy controls, ^S^sham neurostimulation, ^N^non-neurostimulation treatment, ^Z^no treatment.

### 3.2 Clinical outcomes

55 of the 57 primary studies in this review assessed clinical outcomes. 24 of these enrolled a CUD sample, and 31 a MUD sample. 35 employed a sham condition. Given the importance of an adequate control condition to assess change in clinical outcomes from interventions, results were organized according to studies with and without a sham condition. The most assessed clinical outcome was craving, although other assessed outcomes included patterns of use, withdrawal symptoms, affective symptoms, cognition, and sleep. **Tables 1.1**, **1.2** provide a comprehensive summary of the studies. Further details on the outcome scales and scores used are included in **Supplementary Table 1**.

#### 3.2.1 Craving

50 studies included a measure of craving, most commonly via Visual Analogue Scale (VAS) or Desires for Drug Questionnaire (DDQ) (19), with 31 including a sham condition (15, 17, 20–48).

##### 3.2.1.1 Studies with a sham condition

20 sham-controlled studies reported a significant decrease in cravings following active neurostimulation treatment compared to sham (15, 21, 23–25, 27–29, 31, 32, 34–37, 41, 42, 44–47), with one of these finding significance only in the craving induction condition and not in instant craving (32). An additional study found a significant reduction in cravings from baseline in the active group only, although comparison was not made directly to sham (17). A further four found an improvement in cravings in the active group, although not statistically significant (26, 30, 39, 43), three found changes in both active and sham groups (22, 33, 38), and finally three reported no change at all in craving measures (20, 40, 47). One study found the sham group to have a greater reduction in cravings than the active group (48). One of the studies that reported an non-significant change in the active group compared to sham estimated that significance would be reached if a sample size of more than 15 per group was enrolled (26).

##### 3.2.1.2 Studies without a sham condition

The remaining 19 studies that assessed cravings did not incorporate a sham condition into their methodology (11, 16, 49–65). Three of these studies recruited healthy controls to serve as a comparison group (49, 50, 60). Following neurostimulation, a total of 14, including the three with healthy controls, reported a significant improvement in cravings compared to baseline in at least one active condition (11, 49–55, 57–62), one of these also found a significant reduction in the control group, however overall the group with add on rTMS showed a significantly greater reduction compared to this control (53). and five found a statistically non-significant change (16, 56, 63–65). The only study to investigate whether comorbid psychiatric diagnoses impacted the effectiveness of neurostimulation treatment on craving found no significant difference between CUD participants with or without ADHD, with both groups showing a significant reduction in craving post active treatment from baseline (51).

#### 3.2.2 Patterns of use

Of the 18 studies that reported outcomes related to substance use patterns, ten employed a sham condition (20–22, 25–27, 29, 30, 33, 66). Subjective measures of use, mostly self-reported use quantity, frequency, or number of relapses, formed the majority of analyses. A handful of studies also used urine drug tests to assess this outcome (22, 25, 27, 30).

##### 3.2.2.1 Studies with a sham condition

Two studies found active neurostimulation to significantly reduce use compared to sham (26, 30), with one of these only finding the significant reduction in participants who use crack-cocaine and not in those who use powdered cocaine (30). Active stimulation was also associated with an increase in self-reported readiness to change drug use behaviours compared to sham (26). Conversely, five studies reported a non-significant reduction in use in active compared to sham (20, 21, 25, 29, 66), although one of these reported a significant reduction in the active group compared to baseline (66). Three saw a variable improvement in both active and sham groups (22, 27, 33).

One study found that 9.6% of sham group participants relapsed compared to 3.1% of active iTBS group participants at three-month follow-up post-discharge from a rehabilitation facility, although this difference was non-significant (29).

##### 3.2.2.2 Studies without a sham condition

A further eight studies assessed drug use behaviours without a sham condition (51, 52, 54, 56, 57, 62, 65, 67), of which six reported a significant improvement post neurostimulation (51, 52, 54, 57, 62, 65). One of these reported weekly consumption in days of use to be reduced by 70% post neurostimulation, whilst also noting a self-reported reduction in compulsive cocaine use whereby participants found it easier to terminate drug use after initiating using behaviours (65). This study, the only to report on polysubstance use and its relationship to neurostimulation therapy, also reported a reduction in the use of other substances compared to baseline, including nicotine and alcohol (65).

Two studies reported on maintenance sessions. Of these one study reported that 61 (81%) participants returned consistently negative drug urine tests (defined as three or more consecutive negative tests) after the acute phase of neurostimulation treatment (67). This same study followed up these 61 participants for a total of 12 months, during which some received maintenance neurostimulation whilst others did not. No significant change in use or relapse rate was found between groups during this maintenance phase, however these participants were less likely to drop out of the study compared to the non-maintenance group (67). Additionally, a case report (n=1) documented three relapses over a six-month period, with maintenance sessions administered at four time points across the study (56), though the case study design makes any interpretation difficult.

#### 3.2.3 Withdrawal symptoms

##### 3.2.3.1 Studies with a sham condition

Only four studies assessed withdrawal symptoms, two of which had a sham condition (22, 38), with one of these reporting a significant reduction post-neurostimulation compared to sham (38), and the other finding a change in both groups (22).

##### 3.2.3.2 Studies without a sham condition

Of the studies that assessed withdrawal symptoms without a sham group, one found a significant improvement post neurostimulation relative to baseline (54), and the other a non-significant change post neurostimulation (11).

#### 3.2.4 Affective symptoms

Of the 22 studies assessing affective outcomes, 13 had a sham condition (15, 17, 20, 22, 25–27, 32, 34, 35, 38, 68, 69).

##### 3.2.4.1 Studies with a sham condition

Three studies reported a significant improvement in affective symptoms or self-reported Quality of Life (QOL) post active neurostimulation compared to sham (22, 35, 38). Of these, one study demonstrated improvement in overall self-reported QOL in the active condition compared to sham, though only non-statistically significant differences in the improvement of affective symptoms of depression was found between conditions (35). The active group however did have a significant reduction in anxiety compared to sham (35). An additional study assessed QOL and found an improvement in both active and sham groups (26). This same study found no change in depression or anxiety outcome scores (26). Of the studies that reported a significant improvement in affective symptoms post neurostimulation compared to sham, one found that the reduction improved further in participants who had more than 40 sessions of neurostimulation, and in those who used psychotropic therapy concurrently (22). Another found significant improvement in anxiety symptoms only, with no significant difference in depression observed (38). Three studies found statistically non-significant improvements post neurostimulation across all affective outcome measures compared to sham (15, 32, 68), two additional studies found non-significant improvements in anxiety (20, 34), but reported significant improvements in both active and sham groups (34) or no change (20), in symptoms of depression. Three of the remaining studies found improvements in all affective symptom outcome measures across both active and sham groups (17, 25, 27), and finally one study found a significant improvement in mood regulation in the active group compared to sham, although this was not accompanied by a significant difference in depression or anxiety between groups (69).

##### 3.2.4.2 Studies without a sham condition

Nine studies assessed affective symptoms without a sham condition (11, 49, 51, 52, 54, 56, 57, 59, 61). Six reported significant improvements across all affective outcomes post neurostimulation (11, 49, 51, 52, 59, 61), with an additional study finding overall psychopathological burden, depression, anxiety and anhedonia to be significantly decreased post neurostimulation, although suicidal ideation was only non-significantly improved by treatment (54). A further study found non-significant improvements following neurostimulation treatment compared to baseline (56) and the final study saw improvements in both active and control groups (57).

#### 3.2.5 Cognition

25 studies assessed cognition-related outcomes, including decision-making, response inhibition and attention, amongst others, the specifics of which can be found in **Supplementary Table 1**. Eight of these examined impulsivity (20, 25–27, 31, 60, 61, 70). A total of 19 had a sham condition (20, 21, 23–31, 34, 38, 39, 42, 47, 68–70).

##### 3.2.5.1 Studies with a sham condition

Of the 12 sham-controlled studies that assessed cognitive outcomes other than impulsivity, seven reported significant improvements to at least one cognitive measure in the active group compared to sham (23, 24, 28, 29, 34, 47, 68). One of these also included a combination group, which received tDCS and Mindfulness-Based Strategic Awareness Training (MBSAT) and found significantly improved executive function (EF) in the combination group immediately after treatment and at one-month follow-up compared to either the MBSAT alone or Sham tDCS alone groups (24). An additional study found no difference between the active and sham groups overall, but did report a significant improvement in memory domains in active group participants post-tDCS compared to baseline (21). Another study also reported a significant improvement in learning and memory in active group participants compared to baseline (42). A further study found a non-significant improvement post neurostimulation in active group participants compared to sham (69), and another noted no difference between active and sham groups on reaction time testing (47). The remaining two studies reported no change post active neurostimulation (30, 38).

Six of the eight impulsivity studies included a sham condition (20, 25–27, 31, 70). Compared to sham, active group participants showed significant improvements in two of these studies (25, 31), and no change in another study (20). Two further studies noted improvements in both active and sham groups (26, 27), and the final study reported sham group participants to better impulsivity scores than their active group participant counterparts (70).

##### 3.2.5.2 Studies without a sham condition

Post neurostimulation, one of the four studies assessing cognitive outcomes other than impulsivity reported significant improvements from baseline (16), and three found non-significant improvements (49, 50, 56), although one described “notable changes” (56). Of the two impulsivity studies without a sham condition one found a significant improvement (60) and the other a non-significant improvement post neurostimulation (61).

#### 3.2.6 Sleep

Ten studies assessed changes to sleep or sleepiness, with five of these including a sham condition (25, 26, 29, 34, 38).

##### 3.2.6.1 Studies with a sham condition

Two studies reported a significant improvement in active groups compared to sham (26, 29), whilst one found insignificant improvements (34) and the last saw improvements in both groups (25). In a final study that compared target sites, sleep improvement was significantly greater when both the DLPFC and vmPFC were targeted compared to DLPFC alone (38).

##### 3.2.6.2 Studies without a sham condition

Of the five non-sham studies reporting on sleep outcomes, four found significant improvements post neurostimulation (49, 51, 52, 61) whilst the fifth saw only an non-significant improvement (54). Additionally, one of the studies that reported a significant improvement in sleep post neurostimulation also found the degree of improvement in sleep scores to be positively correlated with the number of sessions of neurostimulation in the preceding days (52).

##### 3.2.6.3 Safety and tolerability

27 studies commented on the side effect profile or tolerability of neurostimulation treatments, all concluding the technology to be safe and well tolerated by participants (15, 20–24, 26, 27, 33–38, 40, 44, 48, 52–55, 57, 61, 62, 65, 67, 68). All except one study reported only mild side effects, the most common being scalp tingling or discomfort, headache and sleepiness. A single study reported a transient neurological event that could not be reliably attributed to neurostimulation itself (65).

#### 3.2.7 Review findings

Overall, across 25 reviews, including 12 systematic reviews, seven of which conducted meta-analysis, similar results were presented regarding the effectiveness of neurostimulation in StUDs. One review commented on the good quality of the included studies (71), however, another reported possible publication bias (72).

Meta-analysis including sham controlled trials supported the efficacy of neurostimulation in improving cravings (72–79) and other clinical outcomes, including affective symptoms, sleep and cognition (72). Furthermore, subgroup meta-analysis found iTBS to be more effective than rTMS (76), and high frequency treatments were superior to low frequency (77, 78). The relationship between the number of sessions and craving reduction is uncertain, with one review finding them to be significantly negatively correlated, with an increased number of sessions resulting in a greater reduction in cravings (75, 79) whilst another found no such relationship (77).

The scoping review focused on a population with SUD and comorbid neuropsychiatric diagnoses and found rTMS to be safe, efficacious, and well tolerated in these patients, except for some scalp discomfort at higher intensities (8). Additionally, three other reviews found no serious adverse events associated with neurostimulation, and an overall low side effect profile (80–82).

Common recommendations across reviews included the need for larger sample sizes and/or longer follow-up periods (74, 81, 83–87). Many also suggested future studies with a focus on optimising protocol parameters (81, 88–93), with a handful of systematic reviews declaring heterogeneity of study stimulation and protocol parameters a barrier to completing a reliable meta-analysis (80, 84). Some suggested further research into neurostimulation mechanisms (88, 91) and determining patient suitability for neurostimulation treatment (94). For a more comprehensive summary of included reviews, see **Supplementary Table 1**.

### 3.3 Comparison of neurostimulation modalities

Three primary studies compared different neurostimulation modalities in relation to clinical outcomes (11, 61, 62) (**Table 2**). Two compared iTBS to rTMS, with neither finding significant differences overall between the two modalities (11, 62), however one found rTMS to significantly improve symptoms of anxiety and depression whilst iTBS did not (11). Neither of the aforementioned studies had a sham condition. The final study explored iTBS in comparison to cTBS, and although no significant difference was noted in clinical outcomes, adverse effects were significantly higher in the iTBS group compared to other groups, with the main issues being tingling and sleepiness (61).

Three reviews assessed for any differences between neurostimulation modalities (6, 95, 96). The greatest improvement in craving scores was observed in participants treated with a combination of iTBS of left DLPFC and cTBS of left vmPFC (6).

### 3.4 Comparison of target sites

Nine studies compared target sites, as seen in **Table 3**. Most of these compared variations of DLPFC targeting, whether left to right, or various electrode montages over this region. Only a single study reported the right DLPFC to be significantly superior (59), with the most recent of the studies finding both left and right DLPFC high-definition tDCS groups to show similar significant reductions in craving compared to sham tDCS, lasting up to one month (37, 58). In addition to these findings, one study reported targeting the left DLPFC but not the right DLPFC, produced a significant improvement in anxiety, whilst targeting either the left or right DLPFC significantly improved depression (61).

In one study comparing various electrode montages, two of the montages were found to be significantly more effective in improving specific cognitive outcome measures (16). Finally, in a study that assessed vmPFC as an additional target site, a greater significant effect on depressive and withdrawal symptoms, as well as sleep, was observed in participants who received neurostimulation targeting both the DLPFC and the vmPFC compared to either site alone (38).

Three reviews comparing target sites were assessed, both reporting the DLPFC as the primary target and finding it effective for reducing cravings in StUDs (97–99).

### 3.5 Neuroimaging

17 primary studies and one review assessed the effects of neurostimulation treatment, accompanied by neuroimaging. A summary of results can be found in **Tables 4.1**-**4.2**. It should be noted that the following neuroimaging findings are promising but preliminary, and that casual inferences should be avoided at this stage.

#### 3.5.1 MRI

Four studies with a CUD patient sample found neurostimulation to alter neural pathways, activity and/or white matter connectivity and organisation between various brain areas, including the DLPFC and vmPFC (20, 25, 39, 64), with one study finding these changes were associated with a significant reduction in cravings which remained at three-month follow-up (25). This effect was lost by the six-month endpoint (25). One of these studies, a sham-controlled tDCS study in people who use of crack-cocaine which employed a combination of EEG and MRI data, found active tDCS improved connections between the vmPFC and nucleus accumbens (64). These changes corresponded to reduced drug cravings clinically (64).

In a study that compared brain activity in CUD patients to healthy controls, the ventral striatum was found to have elevated baseline gradient values in CUD patients (63). This was normalised towards controls following rTMS treatment and significantly correlated with a reduction in craving (63). A final study assessed for changes in both the dorsal and ventral striatum, finding these areas to become less active following active cTBS treatment as opposed to sham (43).

In a study conducted in a MUD sample, connections between the left DLPFC and inferior parietal lobule were found to be improved following active iTBS, but not sham, which correlated with a significant reduction in craving scores (41). Furthermore, the study reported decreased connectivity between the insula and DMN regions after active iTBS, which was suggested to be a potential mechanism for decreased attentional bias towards drug cues (41). An additional two MUD studies also investigated activity in the DMN, alongside some other relevant areas (15, 44). One found that active rTMS resulted in increased activity and communication between the VAN and ECN post neurostimulation, whilst decreased communication was observed between the VAN and DMN compared to sham, with these changes correlating with a decrease in craving (15). The other found similar changes between pre-tDCS and post-tDCS (44).

In contrast, a separate study of 60 MUD patients found greater response to drug cues in the active tDCS group than in the sham group, who showed a decrease in brain activity following cues (40). No significant changes were reported for craving scores between active tDCS and sham groups (40). The final study utilised Proton magnetic resonance spectroscopy (H MRS) to assess for brain changes, and focused on levels of GABA, glutamate and glutamine (Glx) and n-acetyl-aspartate (NAA) (42). Reported findings included significant reductions in GABA/NAA in the active rTMS group compared to sham, with reductions in GABA specifically significantly correlated with improvements in cognitive function (42). Conversely, Glx/NAA levels were decreased in sham participants but not in those who received active rTMS (42).

An additional study used fMRI and machine learning to identify a functional connectivity signature that was somewhat consistent across the brains of CUD patients but distinct from that of healthy controls (100). They hoped this would help inform the target site and may also help predict treatment response to rTMS. The study was able to determine a functional connectivity signature and used this to target appropriate brain areas with either active or sham rTMS, finding that they were able to accurately predict changes in craving levels post-treatment in the active group participants (100).

#### 3.5.2 EEG

Five of the EEG studies enrolled MUD patients, with the remaining study focusing on CUD. The single study that explored EEG changes in response to tDCS in patients with CUD reported that after only a single session of active tDCS, P3 current density increased in the left DLPFC during neutral cues and decreased during crack-related cues (101). The opposite was seen in the sham group. Following repeated active tDCS sessions, P3 current intensities increased in response to crack-related cues (101).

Two of the MUD-focused studies selected iTBS as the intervention, with one study reporting a significant decrease in the theta-to-beta ratio following active iTBS compared to sham (46). The other study reported stronger P3 amplitudes in the active DLPFC iTBS group following exposure to neutral cues, which was found to be connected to a faster response on a task (47). It also described a reduction in beta-wave activity in the frontal lobe of the brain compared to the sham group when drug-associated cues were shown (47). A further two studies looked at tDCS as the intervention of choice (17, 69). One found active tDCS resulted in an increased amplitude of P300, to a significant level for neutral cues, trending towards the healthy controls (69), however, no significant effect was noted on behavioural performance, although an effect nearing significance was seen in the active tDCS group (69). The other found a reduction in P3 amplitude in the active tDCS group in response to drug-related cues, and an increase in the sham group (17). The active tDCS did not affect the late positive potential when compared to sham (17). The final primary study assessing EEG changes reported changes across four predefined microstates (45). Two of these microstates showed lower activity duration in EEGs obtained from MUD patients compared to healthy controls, and following active rTMS, these showed significant improvements, trending towards healthy control activity. Such changes were found to be significantly associated with decreases in craving scores (45).

Only one review was identified that directly assessed neuroimaging (102), reporting that imaging modalities have provided useful insights into neurostimulation mechanisms and addiction pathophysiology, as well as predicting treatment response via fractional anisotropy.

A handful of other reviews recommended imaging studies as a future focus in the research space, although they did not focus their assessment on this.

## 4 Discussion

Despite the ongoing health and societal related burdens of StUDs they continue to lack a reliably effective treatment, with most current regimes relying on psychotherapy and pharmacological withdrawal symptom mitigation, each with varying success rates. Neurostimulation has held its place as a prospective upcoming tool in the management of such disorders, stimulating growth in related research studies. Whilst previous reviews on the topic have been published, many focus on other SUDs, or include a smaller number of articles, and/or outcomes, in their analysis. This review provides a more comprehensive overview of a greater number of studies and offers a unique summary of multiple outcomes of interest when it comes to non-invasive neurostimulation treatment in StUDs specifically. Across the studies consulted in this review, the overall trajectory of non-invasive neurostimulation techniques in the management of StUDs appears promising, although further progress and investigation is still required.

### 4.1 Clinical

A relatively even split of studies with MUD and CUD patient samples were included in this review. Cravings were the most frequently assessed outcome, which is consistent with previously published reviews (90). Two-thirds (66%) of the studies measuring cravings found active neurostimulation therapy to produce significantly better reductions in craving scores than comparator groups, or to significantly reduce baseline levels of cravings. Contrastingly, two studies reported an increase in at least one measure of craving in active groups compared to sham groups (36, 48). Furthermore, as mentioned in the reporting of results, a number of studies found non-significant differences between active and sham groups. However, despite this, craving reduction remains the most reproducible outcome across studies, with most of this evidence coming from sham-controlled trials. In interpreting these results and their reliability it must be noted that all except one study used subjective measures of cravings (37), highlighting a gap in objective craving data, such as heart rate. Findings across other clinical outcomes including use patterns affective symptoms, and cognition are more variable and are frequently reported by methodologically weaker studies.

In terms of follow-up, five studies completed a one-month period (24, 26, 28, 37, 55), three a three-month period (20, 29, 66), two a six-month period (25, 56) and finally a single study followed-up participants for a total of 12-months which was the longest period observed across studies (67). Their outcome of interest was abstinence (67). Reductions in cravings were observed to last up for a maximum of three months after treatment across all reviewed literature (25). This poses the question of how long clinical improvements actually last for following an acute phase of neurostimulation treatment, something that has already been asked and dicsused in previous reviews (89).

This leads into the important real-world application of the role that maintenance neurostimulation sessions play in long term effectiveness of treatment. Although a handful of studies employed maintenance treatment regimes, the volume of data on their efficacy remains extremely limited, and further consensus is required to accurately determine how these can be best optimized. The only study that explored the indications for maintenance therapy in depth was a case report with a sample size of one, reducing its generalisability to broader target populations (56).

Overall results appear to show promise in neurostimulation managing affective symptoms, although the variation in significance of improvements, and scales used, must be acknowledged. Out of all the clinical outcomes assessed in this review, affective symptoms were the most commonly improved in both active and sham groups, potentionally revealing a greater susceptibility to placebo effect (25–27, 34, 57). The most frequently assessed affective outcomes were depression and anxiety. In addition to neurostimulation significantly improving affective symptoms, one of the studies also found this improvement to be even greater compared to sham if participants received more than 40 neurostimulation sessions and/or were taking psychotropics concurrently (22). A similar relationship was found by a study assessing sleep quality improvement, whereby participants who had completed a greater number of neurostimulation sessions in the days preceding the completion of sleep scale scores, experienced greater benefit (52). These correlations could be worth further exploration given it’s clinical application for more holistic and comprehensive regimes.

Cognition was variably affected by neurostimulation therapy, with just over half the studies assessing it finding significant improvements. However, cognitive outcomes were measured with several different tasks, which assessed varied components of cognition, making direct comparisons and conclusions difficult. Although this variation means specific findings are less directly replicable across current literature, it does allow the broad impact of neurostimulation on multiple facets of cognition to be seen. In studies that found significant cognitive improvements in active groups compared to sham, effects were seen to last until as long as one-month follow-up (24, 28). Only a single previously published systematic review included in this narrative review conducted a meta-analysis for cognitive outcomes (72). The effect of neurostimulation on various cognitive tasks assessing episodic and working memory, problem solving and visuospatial learning, amongst other domains, was analysed (72). Significant improvements in the active group participants compared to sham were noted in all but two domains, working memory (P = 0.08) and emotion recognition (p=0.28) (72). Of the domains that were significantly improved by neurostimulation intervention, the degree of overall effect was highly variable, episodic memory (p=0.0007); problem solving, reasoning and visuospatial learning and memory (p=0.001) and visuospatial learning and associations (p=0.02) (72). Although more frequently reaching significance, the variability in the meta-analysis’ findings is in line with the varied effects uncovered in this review. These more convincing meta-analysis findings may also be explained by the stricter inclusion criteria leading to the analysis of only five studies, all of which had to be sham-controlled trials to be eligible for inclusion. Ultimately, it is a reassuring finding that meta-analysis of methodologically strong studies supports neurostimulation in improving cognitive outcomes in MUD patients.

Whilst the above points support potential clinical applicability of neurostimulation in StUD management, it is important to recall that a handful of studies saw clinical improvements in at least one outcome in both intervention and control group participants (17, 22, 25–27, 33, 34, 38, 53, 57), suggesting a degree of placebo effect within this space. Furthermore, only nine studies assessed neurostimulation with current addiction treatment (20, 21, 24, 25, 46, 53, 55, 57, 66), despite this being the most likely way in which the technology would be introduced into the clinical space, bringing into question the extent of generalisability, and the need for future studies assessing combination treatment. Of the studies that did assess both neurostimulation and standard treatment, most found that neurostimulation improves outcomes, with only one study reporting this improvement to be non-significant (66). Although small in number, these studies provide a good starting point and insight into how using neurostimulation as an adjunct to standard therapy might apply to real-world patient management. Furthermore, only a single study explicitly explored the impact of having a comorbid psychiatric diagnosis of ADHD on the effectiveness of neurostimulation treatment in addiction (51). Although this study found no significant differences in the improvements post neurostimulation between participants with ADHD and CUD or CUD alone, there is insufficient evidence to predict whether this is the case with other psychiatric diagnoses at this time.

In general, a trend of good tolerability was observed across all of the studies that commented on this, with mostly only minor side effects reported, tingling of the scalp being the most frequent. This augurs well for the safety of implementing such therapy in the clinical space.

### 4.2 Stimulation types, parameters and protocols

Only a single review study found one neurostimulation type to be superior to another (76), although others highlighted important differences between types and their parameters, which may be of use clinically. iTBS was as effective as traditional rTMS (11, 62), which is important given the practicality of its shorter session times. This may make treatment easier to adhere to, especially given that compliance can be challenging in target populations. Comparisons between high and low frequency have yielded mixed results, with one study reporting no difference in outcomes (58), whilst a review declared high-frequency to be superior (6). Furthermore, multiple treatment sessions produced superior clinical outcomes compared to single treatment sessions (75, 91). Aside from these few direct comparisons between parameters, stimulation protocols were vastly heterogeneous across the studies, making determining which factors produce the most efficacious treatment regime almost impossible at this stage.

### 4.3 Target site and imaging studies

The most established target site is the DLPFC, with most studies finding it an effective target. This can be explained by the known role of the PFC in the pathophysiology of addiction, with changes in its activity as demonstrated by studies employing fMRI, being associated with improved executive functioning and reductions in cravings. The vmPFC has also been addressed as a target site, with its role in emotional regulation and decision-making, justifying this. One of the reviewed studies found that targeting both the DLPFC and the vmPFC was superior in terms of clinical outcomes compared to targeting either site alone (25, 38). This may suggest that having multiple target sites could improve the clinical efficacy of neurostimulation in StUDs, although evidence remains sparse.

Imaging studies that used MRI and/or EEG have assisted in hypothesising and understanding the underlying mechanisms of neurostimulation and the brain regions likely to be most efficacious when targeted, however, they are still relatively small in number when it comes to StUDs and present some conflicting results.

What appears to be very sparse are more studies that frequently compare brain activity in patients to that in healthy controls, although what we have observed so far suggests neurostimulation may be able to normalise patients towards this baseline (45, 63). A fascinating, novel concept identified in this review is individualised montages, and this approach, although still in its early development, has shown promise in not only clinical results but also in predicting response to treatment on a per-patient basis (100). If this develops, it would likely be useful for clinicians in selecting which patients they wish to manage in this way.

EEG studies primarily focused on P3, theta and beta activity, with mixed results, making definitive conclusions difficult to draw. Despite this, all EEG studies have found that neurostimulation alters activity in some way.

### 4.4 Strengths and limitations

The strengths of this review include the number and variety of studies included. It is one of the most extensive reviews on this topic, certainly in StUDs specifically. This meant we were able to assess multiple relevant outcomes and variables when it comes to neurostimulation in the addiction medicine space. However, this review has limitations, including the use of a single reviewer for inclusion and exclusion criteria, and the absence of quality or bias assessments or meta-analyses. As such, it is recommended that readers weigh the results obtained from sham-controlled study designs more heavily than those with less rigorous methods. Furthermore, although our scope adjustment allowed us to assess the literature in the StUDs space more comprehensively, it does mean we could not evaluate the literature available for other SUDs as initially intended.

In terms of the included studies, a growing number of randomised controlled trials were identified, as well as those employing a sham condition, which increases the usefulness of this review. However, only just over half of the studies reporting on clinical outcomes employed a sham, and a handful of other primary studies still failed to even employ a control group, comparing only to baseline levels. Many sample sizes were also relatively small, with most employing under one hundred participants, with more male than female representation. These small sample sizes have been predicted to be the cause behind results not reaching significance, with one study stating significance would be reached should the study have had more participants (26). It was noted that female representation in samples is increasing in more recent publications, however the discrepancy in male to female representation could be a potential hinderance to the generalizability of presently obtained results to female patients. One of the reviews also commented on the possibility of publication bias in the literature thus far (72).

### 4.5 Recommendations for future research

One of the main gaps identified in the literature that would be a valuable focus of future research is the limited generalisability of study samples to the real-world target population. None of the included studies clearly aimed to assessed neurostimulation in patients with diagnosed comorbid affective disorders, which commonly exist in patients with SUDs. Only a single study evaluated outcomes in patients with comorbid CUD and ADHD (51). Furthermore, none of the studies had a sample with polysubstance use diagnoses; the only exception to this was a single study that commented on how the use of other substances was also reduced in their participants who underwent active iTBS for CUD (65).

Additional gaps included a lack of extended follow-up periods, with few studies following participants past one to three months. Given the relapsing and remitting nature of addiction disorders, this is a considerable limitation to the current literature and how it can be applied to a clinical setting. Further research is needed on the duration of results following initial treatment and the role that maintenance therapy should play in the ongoing management of these patients. Ideally, establishing an optimal range of number and frequency of sessions would be highly valuble. As briefly discussed above, the heterogeneity of stimulation parameters was extensive across the reviewed literature (7, 74, 76, 80, 84, 88). As such, more studies that aim to compare various parameters such as frequency, duration and number of sessions may be valuable in eventually constructing future clinical guidelines.

A final area of recommendation would be to further explore the personalised medicine approach and investigate possible predictors of treatment response so that clinicians can better determine patient suitability for this treatment. This area is particularly relevant as medicine moves towards a more individualised patient care model. Data sharing initiatives for non-invasive brain stimulation techniques, such as Big NIBS data (103–105), may help to address field-wide issues associated with low sample sizes and poor statistical power to detect significant effects, as was evidence in some studies included in this review.

### 4.6 Other important considerations

Other important considerations include funding, cost, ethics, and the availability of this treatment, especially considering the social complexities and vulnerability of many patients in addiction medicine clinical practice. Future exploration into the logistics of delivering neurostimulation treatment to vulnerable patients would be of value, including equity of access.

### 4.7 Conclusions

To conclude, this review is a comprehensive overview of the currently available literature on non-invasive neurostimulation in the treatment of StUDs. Whilst neurostimulation is a promising upcoming treatment modality in StUD management, with current results generally positive, at present it should be considered experimental or adjunctive, rather than a standalone evidence-based treatment. We also recognise the variation and occasional contradictions in the research findings and suggest ongoing investigation into these limitations moving forward.

## Appendix A - Search Strategy

**Table A1:** Ovid MEDLINE Search Strategy.

Search	Query	Results
All searches were limited to: from 2003/1/1-2025/4/23 (date of search)		
#1	Substance-Related Disorders/	110,654
#2	Addiction Medicine/	289
#3	(Addiction OR “Addicti* disorder*” OR “substance use disorder*” OR “substance use” OR “substance dependen*” OR “substance misuse” “substance abuse” OR “hazardous substance use*” OR “tobacco use disorder” OR alcoholism OR “alcohol dependen*” OR “alcohol use disorder” OR “stimulant use disorder*” OR “substance-related disorder*” OR “addiction medicine”).mp	294,179
#4	(Cocaine OR Cannabis OR methamphetamine* OR opioid* OR heroin OR alcohol* OR smoking OR tobacco OR nicotine OR stimulant*)	1,034,095
#5	1 OR 2 OR 3 OR 4	1,140,900
#6	(TMS OR “transcranial magnetic stimulation” OR rTMS OR “repetitive transcranial magnetic stimulation” OR tDCS OR “transcranial direct current stimulation” OR “direct transcranial ultrasound” OR TUS OR LIFU OR LIFUS OR FUS OR pUS OR TPS OR pTUS OR neurostimulation OR “deep transcranial magnetic stimulation” OR ECT OR “electroconvulsive therapy” OR “deep brain stimulation” OR DBS OR “noninvasive brain stimulation” OR “brain stimulation” OR “transcranial electrical stimulation” OR tES).mp	100,928
#7	Deep Brain Stimulation/	12,425
#8	6 OR 7	100,928
#9	5 AND 8	3181
#10	(withdraw* OR craving* OR “withdrawal symptoms” OR abstinence OR sobriety OR cessation OR “smoking cessation” OR “addictive behaviours” OR “addiction management” OR “addicti* disorder management” OR “addicti* disorder treatment” OR “addiction treatment”).mp	254,797
#11	9 AND 10	601
