# Supplementary material for: Non-invasive neurostimulation techniques for the treatment of stimulant use disorders
Source: Front Psychiatry. 2026 Feb 4;17:1755441. doi: 10.3389/fpsyt.2026.1755441 (PMC12913103; doi:10.3389/fpsyt.2026.1755441)
Supplement: Supplementary file 1 [file Table1.docx]

Supplementary Material

**Supplementary Table 1 – Extended results of all included studies with clinical outcomes**

| Author, Year | Sample size (n) Participant diagnosis | Intervention | Comparator | Outcomes | Findings | |
| --- | --- | --- | --- | --- | --- | --- |
| Non-review studies | | | | | | |
| Cardullo et al., 2024 (49) | Study 1 (short intervals):  n = 38  18 CUD  20 healthy  Study 2 (long intervals):  n = 45  22 CUD  23 healthy | 2 sessions per day 15 Hz rTMS for 5 consecutive days to left DLPFC | Healthy controls received same rTMS | Craving: CCQ, GSI  Sleep quality: PSQI  Depression and anxiety: BDI-II + SAS  Cognition: Temporal processing (time bisection task) | Significant improvement in clinical symptoms (craving, anxiety, depression and sleep) in the treatment group compared to baseline.  Temporal processing in CUD patients after rTMS normalised towards controls. | |
| Liu et al., 2024 (50) | 89  50 male MUD   - 24 rTMS - 26 no treatment   39 healthy controls | MUD only: 20 days (daily 5 days/week) of rTMS over left DLPFC | No treatment  Healthy controls (for baseline cue-induced craving scores and IGT) | Cue-induced craving  Cognition: Decision-making (IGT) | Significant decrease in craving score between pre- and post-treatment in rTMS group compared to no-rTMS.  Decision-making improved in rTMS group compared to baseline. | |
| McCalley et al., 2024 (20) | 33  17 cTBS (13 completed)  16 sham (12 completed)  CUD | 10 sessions cTBS over left frontal pole of the MPFC across 3 weeks + behavioural counselling | Sham cTBS + behavioural counselling | Cocaine abstinence over 3-month follow-up  Craving: CCQ  Anxiety: STAI  Depression: BDI-II  Impulsivity: Barratt Impulsivity Scale | No significant difference between active and sham on abstinence over time (though abstinence was greater in the intervention group compared to control (1-month 92% vs 66.6%; 2- month 100% vs 66.6% 3-month 85% vs 66.6%).  No change to craving, anxiety, depression or impulsivity. | |
| Fayaz Feyzi et al., 2022 (21) | 60 (43 completed)  15 Active tDCS  12 Sham tDCS + Matrix  13 Matrix only  MUD | 16 sessions of 2 mA left DLPFC tDCS PLUS Matrix Model psychotherapy | Sham tDCS PLUS Matrix Model psychotherapy  OR  Matrix Model psychotherapy alone | Craving: OCDUS  Cognition: Working memory (WMS), executive function - set-shifting (WCST)  Relapse rate | In comparison to both comparator groups, the active tDCS group showed a significantly greater reduction in craving score compared to both sham and psychotherapy alone.  Aspects of cognitive testing performance (visual working memory, auditory working memory, true and false answers in the WCST) were significantly improved in the active tDCS group only compared to baseline. There were no significant differences between groups, though effect sizes ranged from small to large.  Non-significant reduction in relapse rates was observed in the active (18%) and the two control conditions (50% sham, 55% psychotherapy only). | |
| Jiang et al., 2022 (70) | 45  23 Active tDCS  22 Sham  MUD | 2 mA daily tDCS for 5 days over DLPFC | Sham tDCS | Behavioural impulsivity: 2-choice oddball task | Behavioural impulsivity increased from baseline in active tDCS group after 5 days: no difference from baseline in the sham group. | |
| Liu et al., 2022 (60) | 58  29 MUD  29 healthy controls | 5 sessions of HF-rTMS on left DLPFC per week for 4 weeks | Stop signal and NoGo tasks only evaluated in controls (no intervention administered) | Cue induced craving: self-rated following a video on methamphetamine use  Cognition: behavioural inhibition (Stop signal reaction time [SSRT],  Stop-signal Delay [SSD], NoGo Task)  Tolerability: self-report | Intervention group showed significantly decreased craving and SSRT (impulsivity/inhibitory control) compared to baseline.  No change in other behavioural inhibition tests (SSD or NoGo Task) from baseline.  Mild transient side effects only. | |
| Martinotti et al., 2022 (22) | 80 (69 completed)  42 Active rTMS  38 Sham  CUD | 15 Hz 2 daily session for 5 days/week for a total of 20 sessions over 2 weeks of rTMS left DLPFC, followed by 12 weeks of maintenance (1 day/week, twice a day). Follow-up (no rTMS) for 3 months following maintenance. | Sham rTMS | Cue induced craving: CCQ-Brief  Cocaine consumption:  Self-reported days of cocaine use and urine tests  Depressive symptoms: MADRS  Cocaine withdrawal symptoms: CSSA | No significant differences between groups on craving and cocaine consumption (both active and sham rTMS groups showed a significant decrease in craving and consumption from baseline).  Active rTMS group showed significantly greater reduction in depressive symptoms than sham at end of treatment, after maintenance, and at follow-up. An even greater reduction was noted in participants who received > 40 sessions and/or used psychotropic therapy concurrently.  No significant difference was observed between active and sham on withdrawal and positive urine tests (withdrawal symptoms significantly decreased in both groups; number of positive urine tests insignificantly decreased in both groups). |  |
| Sanna et al., 2022 (67) | 89 for the acute phase. 61 drug-free participants at end of acute phase offered maintenance.  27 selected maintenance treatment (19 at 12-month follow-up)  34 did not (14 at follow-up)  CUD | Acute: 20 sessions of bilateral iTBS of PFC. Maintenance: 1 session/week for 1 month followed by 2 sessions/2 weeks for 2 months) | Maintenance vs non-maintenance | Abstinence  Dropout rates | 81% of participants were abstinent after the acute phase.  At 12 months, 69.7% were abstinent, 30.3% relapsed. No significant difference in relapse/use between maintenance and non-maintenance groups at 3, 6, and 12-month follow-up.  Maintenance significantly reduced dropout (30%) compared to non-maintenance (59%). | |
| Wang et al., 2022 (23) | 64  34 Active rTMS  34 Sham (4 dropped out)  MUD | 10-Hz rTMS of left DLPFC 3 times/week for 4 weeks | Sham rTMS | Craving: VAS score  Decision-making: IGT | Active rTMS significantly improved craving compared to sham.  Active rTMS significantly improved on block 4 (adverse selection) and 5 (impulsive decision making) on the IGT compared to sham. | |
| Alizadehgoradel et al., 2021 (24) | 80  20 Active tDCS alone  20 Mindfulness alone  20 Combined  20 Sham  MUD  Youth population 18-21 years | 1.5 mA tDCS 12 sessions alone  over DLPFC  1.5 mA tDCS 12 sessions tDCS over DLPFC PLUS mindfulness (MBSAT) | Mindfulness group (MBSAT)  Sham tDCS group | Executive function (EF; N-back test [working memory], Wisconsin card sorting test (WCST), Balloon analogue risk task (BART) [risky decision-making], Go/No-go [response inhibition]  Craving: DDQ | Both immediately after intervention at 1-month follow-up, performance in most EF tasks was significantly better in the combination group compared to baseline and other groups. Some significant improvements in EF were also seen in either tDCS or mindfulness alone compared to sham (though neither singular intervention significantly outperformed the combined intervention).  Cravings were significantly decreased in all intervention groups compared to sham at post-stimulation and at 1-month follow up. | |
| Cardullo et al., 2021 (51) | 230  22 ADHD + CUD  208 CUD | Twice daily 15 Hz HF-rTMS to left DLPFC for 5 consecutive days, followed by twice daily sessions once per week for 11 weeks. | N/A | Cocaine use: urine drug testing, self-reported use and collateral reports of use  Craving: CCQ  Negative affect symptoms: BDI-II, SAS, SCL-90-R  Sleep: PSQI | Both participants with ADHD and CUD and CUD alone showed significant improvement across all outcomes of interest from baseline, with no significant differences observed between groups. | |
| Garza-Villarreal et al., 2021 (25) | 44 (6 female)  24 Active rTMS  20 Sham  20 underwent maintenance until 6-month follow-up    CUD | Acute phase: 2 daily sessions of 5-Hz rTMS of left DLPFC for 2 weeks plus standard treatment  Open maintenance: 2 weekly sessions of active rTMS for 6 months plus standard treatment | Acute phase: Sham rTMS | Craving: VAS score and CCQ-Now  Impulsivity: BIS11  Cocaine use: self-reported frequency and grams, urine drug testing  Anxiety, depression, broad psychopathology, and sleep quality | rTMS significantly reduced craving (VAS only) and impulsivity in active vs sham at 2 weeks.  No significant differences in anxiety, depression, psychopathology, and sleep quality between active vs sham (significant improvement in both groups from baseline to 2 weeks.  No significant difference between groups on urine drug test results.  For those who underwent maintenance, craving (CCQ-Now) was significantly reduced at 3 and 6 months compared to baseline. VAS craving was significantly reduced from baseline at 3 months, but not 6 months). No significant effect on urine test results at 6 months compared to baseline. Anxiety, depression, and impulsivity were significantly improved from baseline at 3 months, but not at 6 months. | |
| Gaudreault et al., 2021 (26) | 17  8 Active tDCS  9 Sham (6 completed)  CUD | 15 sessions over 5 weeks of 2 mA tDCS over DLPFC | Sham tDCS | Self-reported craving alongside CCQ, OCCS  Anxiety: HAMA  Depression: HAMD  Quality of life: WHO QOL  Sleepiness: Epworth Sleepiness Scale, PSQI and interview  Neuropsychological assessments of verbal and nonverbal IQ, verbal fluency, and temporal discounting/impulsivity  Readiness to change drug use: Contemplation Ladder | Non-significant trend of decreased self-reported cravings in the active tDCS group compared to sham. Lost at 1 month follow-up.  No significant differences in Quality of Life or impulsivity between active vs sham (improved in both groups from baseline). No significant differences for other neuropsychological assessments.  Significant reduction in sleepiness and increased sleep hours in active compared to sham. Sleep quality significantly decreased in both groups, however. Readiness to change showed a non-significant increase in the active tDCS group only. Lost at 1 month follow-up.  No significant differences in depression or anxiety were observed. | |
| Lolli et al., 2021 (27) | 62  32 Active rTMS (13 completed)  30 Sham (12 completed)  CUD | 15 sessions of 15 Hz rTMS over DLPFC over 3 weeks | Sham rTMS | Cocaine lapses: twice weekly urine tests (time to urine negativisation)  Craving: VAS (neutral and cue-induced) and CCQ (current)  Cocaine use pattern: self-reports  Impulsivity: UPPS-P Depression: SDQ  Anhedonia (Snaith-Hamilton Pleasure Scale) | High dropout rate.  No significant difference in positive urine drug tests between active and sham groups, though self-reported use was significantly lower in the active group compared to sham.  Cue-induced craving was significantly decreased from baseline to 4-weeks in the active group only (VAS), though current craving (CCQ) significantly decreased from baseline to 4-weeks in the sham group only.  Anhedonia significantly decreased at 4-weeks from baseline for the sham group only.  There were no significant differences between groups in depression and impulsivity scores. For depression, both groups improved from baseline, though scores significantly increased from 4-weeks to 8-weeks in the sham group only. Significant improvements from baseline were observed for some impulsivity facets (negative urgency: active only reduction at 4-weeks and 8-weeks vs baseline; perseverance: sham only at 4-weeks vs baseline). | |
| Alizadehgoradel et al., 2020 (28) | 39  19 Active tDCS  20 Sham  MUD | 10 sessions 2 mA tDCS over DLPFC over 5 weeks | Sham tDCS | Executive functions (EF; (working memory, inhibitory control, cognitive flexibility, risk-taking)  Craving: DDQ  Tolerability: self-report | Significant improvement on all EF tasks in active vs sham immediately post-treatment and at 1-month follow-up.  Significant reduction in craving between active vs sham.  Well tolerated. |  |
| Gómez Pérez et al., 2020 (52) | 87  CUD | 2 sessions per day of 15 Hz rTMS to left DLPFC for 5 consecutive days, followed by 2 sessions per week for 12 weeks | Waitlist group (30-day observation period; n = 10) | Sleep quality: PSQI  Craving: CCQ  Depression: BDI-II  Anxiety: SAS  Symptoms: GSI  Cocaine use: self-report and urine tests | PSQI, cravings, GSI and negative-affect symptoms significantly improved between baseline and 90 days. Degree of improvement in sleep was related to number of rTMS sessions in preceding days.  Self-reported use of cocaine significantly decreased by day 30 in rTMS patients.  No significant changes in any of the outcomes were observed in the waitlist group. | |
| Su et al., 2020 (29) | 126 (106 men)  70 Active iTBS  56 Sham  MUD | iTBS over left DLPFC for 4 weeks (20 daily sessions) | Sham iTBS | Craving: VAS score  Cognitive function: CogState Battery (verbal working memory, problem solving/error monitoring, working memory, spatial working memory, social cognition)  Sleep quality: PSQI  Relapse: urine test | Active iTBS significantly reduced cravings and increased sleep quality compared to sham.  Verbal working memory was significantly improved in active iTBS compared to sham. Problem solving/error monitoring was significantly improved compared to baseline in the active group only (no significant difference compared to sham). Results for other cognitive outcomes were not reported.  At 3 months after leaving the rehab centre, 63 patients performed a urine test, 9.6% (n = 3) relapsed in sham vs 3.1% (n = 1) in active iTBS. | |
| Verveer et al., 2020 (30) | 59  29 Active tDCS  30 Sham  CUD | 10 sessions 2 mA tDCS over DLPFC across 5 consecutive days | Sham tDCS | Cognitive functioning: (Inhibitory control, risky decision-making)  Cocaine use: self-report and urine tests  Craving: Ecological momentary assessments | No significant difference on relapse at 90-day follow-up between groups. Relapse for crack-cocaine specifically approached significance in favour of the active group compared to sham.  No significant difference in craving between active and sham (significant decrease over time observed in both groups).  No significant changes to inhibitory control or risky decision-making were observed between active and sham. | |
| Yuan et al., 2020 (31) | 73 (men)  MUD  37 Active rTMS  36 Sham  33 Healthy controls | 10 sessions of daily 1 Hz rTMS to left DLPFC | Sham rTMS | Impulse inhibition: 2-choice oddball task  Cue-induced craving: VAS Score | Impulse inhibition significantly improved compared to baseline in the active rTMS group only. A corresponding significant decrease in craving from baseline was observed in the active group only. | |
| Anaraki et al., 2019 (32) | 30  15 Active tDCS  15 Sham  MUD | 5 sessions 2 mA tDCS over DLPFC | Sham tDCS | Immediate craving: DDQ  Cue-induced craving: VAS score | Cue-induced craving was significantly decreased in the active tDCS group. No change was seen in immediate craving. | |
| Liu et al., 2019 (53) | 90 (female)  52 Active rTMS  38 Control  MUD | Routine addiction Rehabilitation PLUS  20 sessions of 10 Hz rTMS to left DLPFC | Routine addiction rehabilitation | Craving: VAS score | Add-on rTMS significantly reduced craving at 30 and 60 days compared to controls (both groups demonstrated significantly reduced craving at 30 and 60 days from baseline. No significant reductions were observed in either group from Day 30 to Day 60).  Greater treatment effect was seen in young, high-craving patients. | |
| Pettorruso et al., 2019 (54) | 20, 16 completed  CUD | 15 Hz rTMS to left DLPFC twice a day for 5 days/week across 2 weeks, followed by 2 weeks of maintenance treatment(1 day/week) | N/A | Withdrawal symptoms, craving, anhedonia, suicidal ideation: CSSA  Anxiety: Zung Self-Rating Anxiety Scale and SCL-90  Depression: BDI and SCL-90  Psychopathological distress: SCL-90  Sleep: Insomnia Severity Index | Significant reduction in negative urinalysis compared to baseline (at 4 weeks, 56.25% had a negative urine test).  Significant reduction in cravings at 4 weeks compared to baseline.  Significant reduction in withdrawal symptoms at 2 weeks and 4 weeks compared to baseline.  Significant reduction in overall psychopathological burden, and depression and anxiety symptomology at 2 weeks and 4 weeks compared to baseline.  Significant improvement in anhedonia at 2 weeks and 4 weeks compared to baseline.  Non-significant reduction in suicidal ideation at 2 weeks compared to baseline.  Non-significant improvement in sleep compared to baseline. | |
| Steele et al., 2019 (65) | 19  CUD | 3 sessions per day of iTBS for 10 days over a 2-week period to dIPFC | N/A | Compliance to iTBS  Safety: reporting of side effects  Cocaine use: self-reported and urine test  Craving: CCS + CCQ  Various other self-report and interview-guided measures (statistical change not assessed) | 9 patients completed the study, 10 did not.  Well tolerated, with the main side effect reported being occasional headaches.  1 transient neurological event (unclear aetiology)  1 cocaine-induced psychosis 2 weeks after discontinuation of iTBS  Significant reduction in days of use and money spent on cocaine at 1 week and 4 weeks compared to baseline in those who completed the study.  A reduction in use of other substances (THC and alcohol, but not nicotine) was also observed.  Mean craving scores decreased from baseline to 1-week and increased towards baseline at 4-weeks (significance not assessed). | |
| Klauss et al., 2018 (33) | 35  19 Active tDCS  16 Sham  CUD | Once daily, every second day (total 10) 2 mA tDCS over diPFC for a total of 10 sessions | Sham tDCS | Craving: 5-item Obsessive-Compulsive Cocaine Use Scale  Relapse: followed up for 60 days | No significant difference in craving between groups (craving scores significantly decreased in both groups).  No significant difference in relapse rates between groups. | |
| Su et al., 2017 (34) | 30  15 Active rTMS  15 Sham  MUD | 5 sessions of 10 Hz rTMS to left DLPFC | Sham rTMS | Cue-induced craving: VAS score  Cognitive function: CogState Battery (verbal learning and memory, problem solving/error monitoring, working memory, spatial working memory, social emotional cognition)  Depression: HAMD-17  Anxiety: HAMA-14  Sleep: PSQI | Craving was significantly reduced in the active rTMS group compared to sham.  Verbal learning and memory was significantly improved in active rTMS compared to sham. Social emotional cognition was improved in the active group compared to baseline, with no difference from baseline observed for sham (between-groups comparison not reported), No significant changes in either group from baseline were observed for problem solving/error monitoring, working memory, and spatial working memory.  Depression: No significant difference between groups (scores significantly decreased in both groups from baseline).  No difference between groups on anxiety or sleep (no change from baseline for either group). | |
| Bolloni et al., 2016 (66) | 10  6 Active rTMS  4 Sham  CUD | 12 10 Hz rTMS sessions, 3 times/week for 4 weeks over PFC PLUS weekly psychological support | Sham rTMS PLUS weekly psychological support | Cocaine intake (ng/mg): hair analysis | No significant difference in intake between active and sham across time. A significant reduction in intake detected in hair samples was noted at 3 and 6 months compared to baseline in the active group. | |
| Rapinesi et al., 2016 (55) | 7 (male)  CUD | 3 sessions of alternate day 20 Hz dTMS over DLPFC, for a total of 12 sessions over 1 month PLUS prior drug treatment | N/A | Craving: VAS score | Cravings were significantly reduced up to 8 weeks following treatment compared to baseline. Cravings significantly increased (towards baseline) from four weeks to eight weeks. | |
| Shariatirad et al., 2016 (56) | 1  MUD | 20 sessions of 2 mA tDCS over right DLPFC at 5 days/week for 4 weeks.  Booster tDCS sessions during 6-month follow-up period if symptoms suggested it was required - intense or frequent cravings, or single episodes of craving that was hard to control | N/A | Craving: DDQ  Substance related symptoms: LDQ  Relapse: urine tests and self-reporting  Cognition: CAQ  Depression: BDI | Reductions in craving, substance-related symptoms, and depression symptoms were observed at 6 months compared to baseline. Scores on memory, inhibitory control and selective attention, decision making, planning, sustained attention, cognitive flexibility, and total number of cognitive tasks were higher at 6 months from baseline, with lower scores observed for social cognition between baseline and 6 months.  3 lapses reported across the 6 months.  4 booster sessions were required on days 67, 70, 72, 88. | |
| Batista et al., 2015 (35) | 36 (male)  17 Active tDCS  19 Sham  CUD | 5 sessions of 2 mA tDCS over 2 weeks | Sham tDCS | Craving: Obsessive Compulsive Cocaine use scale  Depression: Hamilton Depression Rating Scale  Anxiety: Hamilton Anxiety Rating Scale  Quality of life: WHO questionnaire (WHOQOL-BREF) | Craving was significantly decreased in the active group compared to sham. This decreased linearly over 4 weeks, with significant decrease maintained at 1-week post-stimulation.  Depression:  No significant difference between active vs sham (active sig decrease from baseline, sham non-sig decrease from baseline).  Anxiety:  Significant difference between active vs sham (active non-sig decrease from baseline, sham non-sig increase from baseline).  Overall perception of quality of life and health:  Significant improvement in the active compared to sham group. | |
| Terraneo et al., 2016 (57) | 32  Stage 1:  16 Active rTMS  16 Pharmacological (13 completed)  Stage 2:  Active rTMS (10 from pharmacological stage 1 + 15 from rTMS stage 1)  CUD | Stage 1:  Single session of 15 Hz rTMS to left DLPFC  Stage 2:  10 sessions of 15 Hz rTMS to left DLPFC | Stage 1:  Pharmacological agents  Stage 2:  10 sessions of 15 Hz rTMS to left DLPFC for participants managed with pharm in Stage 1 | Cocaine use: urine tests  Craving: VAS score  Depression: SCL-90 | Stage 1:  Significantly more negative urine tests in the rTMS group compared to the control.  Craving was significantly lower in the rTMS group compared to control.  Stage 2:  10 patients (allocated as controls for Stage 1) received rTMS in Stage 2. They showed significant improvement in negative urine tests and craving compared to Stage 1 control, comparable to the rTMS group in Stage1.  No difference in depression scores between groups (observed non-sig increase pre-post in both groups). | |
| Shahbabaie et al., 2014 (36) | 31 (male)  MUD | Single 2 mA tDCS over right DLPFC | Sham tDCS (same participants, crossover) | Craving: VAS score | Significant reduction in at-rest craving was noted at 10 minutes of active tDCS compared to sham. However, an increase in cue-induced craving in active tDCS vs sham was seen, indicating a state-dependent effect of tDCS. | |
| Li et al., 2013 (48) | 10 MUD  8 Healthy controls | 1 session of 1 Hz rTMS over DLPFC | Sham rTMS (crossover design with 1 hour washout) | Cue-induced craving: VAS score | Craving scores were increased after active rTMS compared to sham. No effect on craving was seen in the healthy controls. | |
| Non-reviews: clinical outcomes reported but focus was target site | | | | | | |
| Hou et al., 2025 (37) | 60  MUD | HD-tDCS over left or right DLPFC | Sham HD-tDCS | Craving: Explicit (VAS), implicit (SC-IAT) Physiological craving: HR, BP | Significant decreases in explicit and implicit craving in both active HD-tDCS groups from baseline up to 1-month follow-up compared to sham. No difference between active groups.  Significant reduction for HR in active groups compared to sham post-treatment (no significant differences between left and right DLPFC). No significant differences for BP changes between groups. | |
| Rezvanian et al., 2022 (16) | 15 males  MUD | 2mA tDCS for 20 mins (6 different montages (target site and anode/cathode placement), crossover design | Each electrode site protocol vs the other with a 72-hour washout between them. | Craving  Cognition: Executive functions (inhibition [Go-NoGo), working memory [N-Back task]) | All six protocols significantly increased cognitive inhibition from baseline (most reduction in the left anode/right cathode DLPFC protocol) and presentation error. Elimination error and response time were not significantly different from baseline in any protocol.  Non-significant decreases in craving observed. | |
| Chen et al., 2020 (38) | 74  MUD | Group A: iTBS over DLPFC  Group B: cTBS over left vmPFC  Group C: iTBS + cTBS over DLPFC and vmPFC respectively | Sham TBS | Cue-induced craving: VAS  Depression: HAMD-17  Anxiety: HAMA-14  Sleep: PSQI  Cognition: CogState Battery (verbal learning & memory [ISL], problem-solving/error monitoring [GML], working memory [TWOB], spatial working memory [CPAL], social cognition [SEC])  Withdrawal symptoms: AWQ | Significant improvements in withdrawal symptoms in Group C vs sham.  No significant difference on sleep quality between any active group vs sham, though Group C had improved sleep quality compared to Group A.  Significant improvement in anxiety in Group C compared to sham (no differences in other groups). No significant differences on depression between any groups or compared to sham.  All active TBS conditions significantly and similarly reduced craving compared to sham.  No effect was seen in any group on cognition. | |
| Shahbabaie et al., 2018 (68) | 90  MUD | tDCS (2mA, 13 mins on, 20 mins off, 13 mins on, participants received one of 5 active electrode montages targeting the left/right DLPFC) | Sham tDCS | Attentional bias (Pictorial Probe Detection Task)  Mood: (Depression, anxiety, and stress; DASS-21)  Positive and Negative Affect: PANAS | Active tDCS (left DLPFC/right shoulder and left DLPFC/right DLPFC significantly reduced attentional bias towards drug cues compared to sham.  No significant changes to positive or negative affect or mood between groups. | |
| Liu et al., 2017 (58) | 50  MUD | rTMS (1 session/day for 5 days. Four intervention conditions: 10 Hz or 1 Hz, left or right DLPFC) | rTMS over varied target site and/or varied frequency; Active sham rTMS over P3 | Cue-induced craving (VAS) | No significant difference between either 10 Hz left or right DLPFC when compared to active control over time. There was a significant reduction over time between both the 1 Hz left and right DLPFC when compared to control.  All four intervention rTMS conditions significantly reduced cue-induced craving after session 1 and session 5 vs baseline. There were no significant differences between the four intervention conditions. There was no significant difference over time for the control condition. | |
| Camprodon et al., 2007 (59) | 6  CUD | rTMS (one session, 10 Hz, left vs right DLPFC, crossover design) | N/A | Craving: VAS  Anxiety: self-report (VAS)  Mood: self-report: Happiness vs sadness (VAS) | Right DLPFC rTMS significantly reduced craving at post-stimulation compared to both baseline and left DLPFC stimulation, however this disappeared after 4 hours (no difference from baseline for left DLPFC stimulation).  No significant differences between groups for anxiety, happiness, or sadness, though significant reduction in anxiety and increase in happiness post-stimulation vs baseline for right DLPFC stimulation only (disappeared at 4 hours). Sadness significantly increased from baseline vs post-stimulation for left DLPFC only (disappeared at 4 hours). |  |
| Non-review studies: clinical outcomes reported but focus was differences between neurostimulation modalities | | | | | | |
| Liu et al., 2022 (11) | 20  MUD | iTBS (12 sessions, left DLPFC | rTMS (12 sessions, 10 Hz, left DLPFC) | Cue-induced craving: VAS  Anxiety: SAS  Depression: SDS  Withdrawal symptoms: Withdrawal Symptom Scale for MA Addicts | Both modalities significantly and similarly improved cravings from baseline on days 10, 15, and 20.  rTMS and iTBS significantly improved withdrawal symptoms from baseline to day 20 only (not days 10 or 15; no significant difference between groups).  Significant improvement in anxiety from baseline for rTMS on days 15 and 20, but not day 10. There was no significant difference on anxiety in iTBS from baseline. Overall, there was no significant difference between rTMS and iTBS groups.  Significant improvement in depression from baseline for rTMS on day 20 only. iTBS had no significant change on depression from baseline. Overall, there was no significant difference between rTMS and iTBS |  |
| Zhao et al., 2020 (61) | 83  MUD | 2 day/5 days:  iTBS over left DLPFC  cTBS over right DLPFC | cTBS over left DLPFC (active control) | Cue-induced craving: VAS  Sleep: PSQI  Depression: BDI, HAM-D  Anxiety: BAI  Impulsivity: BIS-11  Adverse effects: Self-report | Significant and similar reduction in craving from baseline with both intervention modalities (no significant reduction for the active control from baseline).  Significant and similar improvement in depression for all groups.  Significant and similar improvement in sleep for both left DLPFC groups (iTBS and cTBS) only from baseline.  Significant improvement in anxiety in iTBS group only.  Adverse effects higher in iTBS.  No effects of any group on impulsivity. |  |
| Sanna et al., 2019 (62) | 47  CUD | iTBS (20 sessions over 4 weeks, 15 Hz (3 mins), bilateral PFC and insular cortex) | rTMS (10 sessions over 4 weeks, 15 Hz (15 mins), PFC) | Craving: CCQ-Brief  Complication from drug use likelihood: Modified WHO-ASSIST  Cocaine consumption: Self-report and urine test | Both conditions significantly reduced use, craving, and risk from baseline - no significant difference between groups. |  |
| Non-review studies: clinical outcomes reported but focus was neuroimaging changes | | | | | | |
| MRI | | | | | | |
| Zhang et al., 2025 (63) | Discovery cohort:  41 CUD  44 controls  Independent cohort:  53 CUD  45 controls  Treatment cohort:  44 CUD  -24 Active  -20 Sham | rTMS (10 sessions, 5Hz, left DLPFC) | Sham rTMS (treatment cohort only)  Healthy controls only used for their fMRI findings | Treatment cohort: Cravings: VAS | Treatment cohort: rTMS reduced cravings. This reduction was significantly correlated with changes to ventral striatal gradients. |  |
| Rasgado-Toledo et al., 2024 (39) | 50  CUD | rTMS (2 sessions/day for 10 days over 2 weeks to left DLPFC) | Sham rTMS | Craving: VAS, CCQ-Now  Impulsivity: Barratt Impulsivity Scale-11 (not directly tested, just predictive modelling) | Reduction in cravings post rTMS compared to baseline.   - Predictive models found this reduction to be significantly greater when baseline intra-cellular volume fraction was low in white matter tracts between caudate nucleus and substantia nigra and pallidum. | |
| Ekhtiari et al., 2022 (40) | 60  MUD | tDCS (1 session, 2mA, 20 mins of right DLPFC) | Sham tDCS | Craving: VAS and DDQ | No significant differences in craving for active vs sham. | |
| Soleimani et al., 2022 (15) | 15  MUD | tDCS (1 session, 2mA, 20 mins of DLPFC) | Sham tDCS | Craving: VAS  Affect: PANAS | Cravings significantly decreased in the active rTMS group compared to sham.  Non-significant changes to positive or negative affect. | |
| Su et al., 2020 (42) | 50  MUD | rTMS (20 sessions over 4 weeks of left DLPFC) | Sham rTMS | Cognitive function: CogState Battery (verbal learning & memory [ISL], working memory (TWOB], problem solving/error monitoring [GML], spatial working memory [CPAL], social cognition [SEC])  Craving: VAS | Significant improvement in ISL in active vs sham. GML scores significantly decreased in active only from baseline (no change for sham). Results on other cognitive tasks not reported.  Significant reduction in craving in active vs sham. |  |
| Su et al., 2020 (41) | 60  MUD | iTBS over DLPFC | Sham rTMS | Cue-induced craving (VAS) | Significant reduction in craving compared to sham. |  |
| Kearney-Ramos et al., 2019 (43) | 19  CUD | cTBS over mPFC | Sham cTBS (same participants - cross over study) | Drug cue reactivity (striatium network)  Craving | No overall significant differences to drug cue reactivity between active and sham. Statistically distinct responses were identified in identified baseline striatum network cue reactivity subgroups (cue-sensitive and cue-insensitive in active group) compared to sham. | |
| Shahbabaie et al., 2018 (44) | 15  MUD | 1 session of 20 min 2mA tDCS over DLPFC | Sham tDCS (same participants - crossover  after 1 week washout) | Craving | Craving decreased significantly in active tDCS compared to sham. | |
| EEG | | | | | | |
| Li et al., 2024 (45) | 34 MUD  17 Healthy controls (no intervention) | rTMS (2 sessions/week for 4 weeks, 10 Hz over left DLPFC) | Sham rTMS | Craving: VAS | Significant reduction in craving level in the active group only from baseline. |  |
| Chen et al., 2023 (69) | 19 MUD (amphetamine)  11 Healthy controls (no intervention received) | tDCS (two sessions of 13 mins separated by a 20 min break, once/day for 5 days, 2mA, DLPFC) | Sham tDCS | Cognition: Behavioural performance (disengagement measured via Posner cuing task variation); Graphical reasoning (Raven’s Graphical Reasoning Scale)  Behavioural Inhibition Scale  Negative Mood Regulation Scale  Anxiety: Situation-Trait Anxiety Scale | Significant reduction in disengagement score (pre-post) for active compared to sham. No effect on graphical reasoning.  No significant differences between active vs sham on behavioural inhibition, negative mood, or anxiety.  Significant improvement in mood regulation in active tDCS group. | |
| Khajehpour et al., 2022 (17) | 42  MUD | tDCS (1 session, 2mA, 20 mins of DLPFC) | Sham tDCS | Craving: VAS  Affect: PANAS | Significant reduction in craving from baseline in the active group only (no between-groups comparison).  Significant reductions in negative affect only in both groups. | |
| Wen et al., 2022 (46) | 15  MUD | iTBS of DLPFC | Sham iTBS | Craving: VAS | Significant reduction in craving in the active iTBS group compared to sham. | |
| Chen et al., 2021 (47) | 49  MUD | iTBS to DLPFC for 20 sessions | Sham iTBS | Cognition: Attention bias (Addiction Stroop Task)  Craving: VAS | Reduced error rate (MUD-related words) in the active group compared to sham. No significant differences between active vs sham on reaction time or error rate for neutral trials.  Significantly reduced craving in active vs sham over time. | |
| MRI and EEG | | | | | | |
| Nakamura-Palacios et al., 2016 (64) | 14 inpatients  9 outpatients  CUD | tDCS (1 session/day for 5 days, 20 mins or 2x 13 min sessions separated by 20 min break, DLPFC) | Sham tDCS | Craving: Brief scale adapted from the OCCUS | Reduction in craving. |  |
| Review studies | | | | | | |
| Author, Year  Type of review | Sample size (n) | Number of studies in review | Types of studies | Focus of review | Findings/implications/gaps/future | |
| Ballester et al., 2024 (90)  Scoping review | 584 | 8 | RCTs | Effect of TMS over DLPFC on cravings | Most consistent finding was a reduction in cravings.  Future recommendations:  Expand clinical outcomes of interest, with more studies focusing on use.  Ensure samples are representative of the general target population as most of the current studies focus on hospitalised patients.  Need more studies with a sample which has comorbid mental health conditions.  Optimise protocol parameters  Increase the number of neuroimaging studies. | |
| Chan et al., 2024 (73)  Systematic review and Meta-analysis | 1095 tDCS and 913 sham (multiple substances not just stimulants) | 43 | RCTs with sham condition | Effect of tDCS on craving in substance addiction generally | tDCS led to a notable reduction in cravings for methamphetamine compared to sham. This was more pronounced when bilateral stimulation was used.   More studies are required to refine stimulation parameters and evaluate long-term efficacy. | |
| Zhang et al., 2024 (91)  Literature review | Not available | Not available | Not available | TMS for the treatment of MUD clinical outcomes such as craving, sleep and mood | Reported mixed results on craving, appears to be with more sessions the more significant the decrease  Discussed advantages of iTBS and cTBS - short duration, safety, tolerability.   Reported improvements in mood post TMS on various anxiety and depression scales, as well as sleep quality.   Focus more on mechanism and honing protocol. | |
| Amerio et al., 2023 (80)  Systematic review | 192 | 8 | RCTs | TMS in cocaine addiction efficacy and tolerability | Best findings were reported by RCTs that used high-frequency (5+ Hz) multiple sessions of rTMS over left DLPFC.  Well tolerated. No serious events. Mild headache.  Studies are still very heterogeneous. | |
| Chmiel et al., 2023 (71)  Narrative review | 243 | 9 | 8 RCT  1 open label | tDCS in cocaine addiction | Consistent decrease to cravings following tDCS.  Relapse rates lower in tDCS groups in 2 studies.  Various studies showed improvements to QOL.  Mixed results on depressive symptoms.  Need more data on brain activity  - fMRI and EEG studies still limited  To increase understanding of cognitive effects maybe combine intervention with cognitive training.  Reviewed studies were deemed to be of good quality. | |
| Guaiana et al., 2023 (74)  Systematic review and meta-analysis | 220 | 6 | RCTs or pre-post design trials | Efficacy and tolerability of tDCS in MUD | Results from craving favoured active tDCS  Tolerability data showed that tDCS does not cause more tingling or itching than sham.  Heterogeneity across studies was high.  Future suggestions:  Larger sample sizes and longer follow-up durations.  More data on attrition rate  Incorporate outcome measures that may more accurately reflect real world clinical scenarios such as days of abstinence or treatment retention. | |
| Mehta et al., 2024 (75)  Systematic review  Meta-analysis was only on alcohol and tobacco studies | 746 stimulants | 6 cocaine  8 MUD | RCTs | Clinical outcomes of tDCS on substance use disorders. | tDCS produced medium effect sizes for drug use and craving.  Multi session protocols were more effective than single sessions. Although the total number of sessions required is still yet to be determined.  Right anodal DLPFC stimulation appeared to be most efficacious.  Heavy male dominance in samples  Future studies should determine underlying neural mechanisms of neurostimulation and further evaluate long-term outcomes and role of maintenance. They should also include study samples with comorbid psychiatric diagnoses.   Individualised target locations is a new area of promise.   Many studies were preliminary (sample <40). | |
| Tang et al., 2023 (8)  Scoping review | Not Available | 274 (48 stimulants) | RCT, open label, secondary analysis, case series, retrospective cohort, prospective cohort | Use of rTMS in SUD for patients with co-existing neuropsychiatric disorders - efficacy and safety | rTMS appears safe in these people.  Gap in the literature is how varying degrees of substance use alters safety, efficacy and mechanism of rTMS.  Some studies had to reduce stimulation intensity due to scalp discomfort.   Improvements in anxiety, depression and insomnia noted in active vs sham. | |
| Tang et al., 2023 (72)  Systematic review and meta-analysis | 608 | 13 (10 publications) | RCTs, thesis | Psychological effects of rTMS on MUD patients | Results of the meta-analysis showed that rTMS was effective in significantly reducing craving scores, improving depression and sleep scores, although no significant effect on anxiety scores was seen.  In terms of cognitive function, there were improvements across many domains, although no effect was seen on social emotional cognition task (SEC) and two back task (TWOB).  Risk of bias was low-unclear across all studies.  Degree of publication bias. | |
| Chang et al., 2022 (76)  Systematic review and meta-analysis | 462 | 7 | DB-RCTs | Efficacy of rTMS in MUD patients | rTMS significantly reduced craving scores compared to sham.  A subgroup meta-analysis found iTBS to be more effective than 10-Hz rTMS.  Significant heterogeneity was observed.  Still lacking long-term studies | |
| Gay et al., 2022 (79)  Meta- analysis | Not available | 34 (12 stimulant) | RCTs | rTMS as treatment for craving in stimulant drugs and behavioural addiction (gambling, eating disorder) | A persistent small effect of neurostimulation was found for stimulant disorder groups. In these groups there was no difference in results identified between different stimulation parameters, however a significant correlation between number of sessions and craving reduction was seen. | |
| Li et al., 2022 (87)  Literature review | 112 (neurostimulation studies) | 3 had neurostimulation as the intervention | RCTs | Treatment of psychostimulant disorder - all treatments from medications to rTMS | Brief courses of 5-10 sessions showed efficacy in reducing withdrawal symptoms, however longer treatment regimens showed improvement to other symptoms such as craving, depression, anxiety and sleep. | |
| Antonelli et al., 2021 (7)  Literature review | Not available | 10 CUD | Not available | Efficacy of TMS is drug addiction | The number of clinical studies remains limited, however of those that have been published, repetitive TMS produces encouraging results, supporting its utility in the treatment of SUD.  Discrepancies exist amongst protocols, e.g. type of neurostimulation, outcome measures and scoring tools, number of sessions and equipment frequencies. Despite this, promising evidence exists for widespread reductions in craving and consumption-reducing effects of rTMS in patients addicted to cocaine. | |
| Torres-Castaño et al., 2021 (81)  Systematic review | 463 | 12 | RCTs, nRCTs, case-series | TMS for treating cocaine addiction - safety, cost and effectiveness | No identified study reported data on cost-effectiveness.  Significant reduction of cravings and use after TMS.  No serious adverse effects were observed. Drop out was not reported to be related to tolerability concerns.  Despite the low quality of the studies the effect of treatment observed is still considered moderate. Some concerns of bias.  Future recommendations:  Larger sample sizes and longer follow-ups are required.  More comparison between stimulation parameters to optimise protocols. | |
| AshaRani et al., 2020 (82)  Systematic review | 222 (neurostimulation as intervention) | 44 (5 neurostimulation) | RCT (neurostimulation studies) | Non-pharm interventions for MUD | The RCTs that assessed rTMS in treating MUD found the treatment to be effective. Effects lasted 30-60 days.  The studies which assessed tDCS also found it to be effective at reducing cravings and improving executive functioning  No serious adverse events were noted. | |
| Ma et al., 2019 (77)  Systematic review and meta-analysis | Not available | 16 | Not available | NIBS on stimulant craving in cocaine, amphetamine and methamphetamine users | The meta-analysis found a significantly strong effect of NIBS on reducing craving levels. The relationship between number of sessions and reduction in craving was not significant.  Subgroup analysis found that only high frequency rTMS resulted in a significant decrease in craving. | |
| Bolloni et al., 2018 (89)  Literature review | 102 | 6 | Unclear | TMS for treating CUD.  Focus on the protocol of stimulation applied including coil type. | Variability in stimulation protocols makes it difficult to draw firm conclusions.   Future studies should focus on determining optimised parameters. | |
| Coles et al., 2018 (86)  Literature review | 153 (stimulant) | 60  4 CUD  3 MUD | Mixed | Brain stimulation to treat SUDs | rTMS and tDCS can have beneficial effects on drug craving and consumption.  Future recommendations for studies:  Have longer treatment periods  Have participants with polysubstance use. | |
| Kedzior et al., 2018 (78)  Systematic review with preliminary meta-analysis | 15 | 9 (2) CUD | RCT and open label | dTMS in SUD | Cocaine craving was reduced after neurostimulation.   High frequency > low frequency | |
| Lapenta et al., 2018 (88)  Literature review | 75 stimulant | 3 stimulant | Mixed | tDCS in addiction and impulse control disorders | tDCS interventions hold several promising clinical avenues in addiction and impulsive control.  Stimulation parameters are very heterogeneous, and future studies should focus on optimising these.  Further investigation into neuroimaging and personalised treatment could be of use clinically. | |
| Rachid, 2018 (83)  Literature review | 149 | 10 | Mixed | Neurostimulation in CUD | Results are promising.  Future recommendations:  Larger samples  Longer studies  Compare stimulation parameters. | |
| Lupi et al., 2017 (84)  Systematic review | 98 (cocaine) | 18 | Not stated | tDCS in SUDs | tDCS resulted in decreased craving, risky behaviours and anxiety. It was also found to  improve quality of life.  Studies were quite heterogeneous.  Future recommendations:  Larger sample sizes  Longer study durations  Optimise parameters and treatment course. | |
| Hone-Blanchet et al., 2015 (94)  Literature review | 95 (stimulant) | 5 | RCT, crossover | Neurostimulation in SUDs | More research needs to focus on stimulation parameters and target sites. | |
| Bellamoli et al., 2014 (85)  Literature review | 42 CUD | 11 (2 CUD) | Not stated | rTMS in the treatment of drug addiction | Future studies should focus on employing multiple rTMS sessions across larger sample sizes with longer follow-up periods. Control group is important. | |
| Gorelick et al., 2014 (93)  Literature review | 58 | 3 CUD  1 MUD | Not stated | TMS in SUDs | Many aspects of optimal parameters are yet to be determined. Future studies should compare parameters including different coil types.  Evidence from depression treatment shows that the total number of rTMS pulses is positively associated with efficacy. Thus far, no addiction study has looked for this association. | |
| Barr et al., 2011 (92)  Literature review | 42 | 2 | Not stated | rTMS for SUDs | HF-rTMS to the DLPFC is effective in reducing the level of cravings for smoking, alcohol, and cocaine.  Further optimisation of stimulation parameters is required. | |

Diagnosis abbreviations: CUD (Cocaine Use Disorder), MUD (Methamphetamine Use Disorder), SUD (Substance Use Disorder)

Brain region abbreviations: DLPFC (dorsolateral Prefrontal Cortex), MPFC (Medial Pre-frontal Cortex), PFC (Prefrontal Cortex)

Technology abbreviations: rTMS (Repetitive Transcranial Magnetic Stimulation), tDCS (Transcranial Direct Current Stimulation), iTBS (intermittent Theta-Burst Stimulation) HF (High Frequency), fMRI (functional Magnetic Resonance Imaging), EEG (Electroencephalogram), NIBS (Non-Invasive Brain Stimulation)

Outcome assessment abbreviations: CCQ (Cocaine Craving Questionnaire), GSI (Global Severity Index), PSQI (Pittsburgh Sleep Quality Index), BDI-II or BDI (Beck Depression Inventory), SAS (Self-rating Anxiety Scale), IGT (Iowa Gambling Task), STAI (State Trait Anxiety Inventory), OCDUS (Obsessive-Compulsive Drug Use Scale), WMS (Wechsler Memory Scale), WCST (Wisconsin Card Sorting Test), SSRT (Stop Signal Reaction Time), SSD (Stop Signal Delay), MADRS (Montgomery-Asberg Depression Rating Scale), CSSA (Cocaine Selective Severity Assessment), VAS (Visual Analogue Scale), SCL-90-R or SCL-90 (Symptom Checklist-90), BIS-11 (Barratt Impulsiveness Scale), OCCS (Obsessive Compulsive Cocaine Scale), UPPS-P (UPPS-P Impulsivity Scale), SDQ (Strengths and Difficulties Questionnaire), DDQ (Desires for Drug Questionnaire), CCS (Cocaine Craving Scale), HAMD-17 (Hamilton Rating Scale for Depression, 17 item), HAMA-14 (Hamilton Anxiety Rating Scale, 14 item), LDQ (Leeds Dependence Questionnaire), CAQ (Cognitive Abilities Questionnaire), WHOQOL-BREF (World Health Organization Quality of Life Questionnaire), SC-IAT (Single Category Implicit Association Test), AWQ (Amphetamine Withdrawal Questionnaire), DASS-21 (Depression Anxiety Stress Scale), PANAS (Positive Affect Score), PAVAS (Negative Affect Score), SEC (Social Emotional Cognition Task), TWOB (Two Back Task)

Study design: RCT (Randomized Controlled Trial), DB-RCT (Double-Blind Randomized Controlled Trial)
