# Supplementary material for: Non-invasive neurostimulation techniques for the treatment of stimulant use disorders
Source: Front Psychiatry. 2026 Feb 4;17:1755441. doi: 10.3389/fpsyt.2026.1755441 (PMC12913103; doi:10.3389/fpsyt.2026.1755441)
Supplement: Supplementary file 2 [file Table2.docx]

Supplementary Material

**Supplementary Table 2 – Extended results of all included studies with a focus on comparing neurostimulation modalities**

| Author, Year | Sample size (n)  Participant diagnosis | Intervention/type of neurostimulation | Comparator | Outcome of interest | Findings |
| --- | --- | --- | --- | --- | --- |
| Non-review studies | | | | | |
| Liu et al., 2022 (11) | 20 (male)  10 iTBS  10 rTMS  MUD | iTBS 50 Hz 80% active motor threshold | 10 Hz rTMS | Cue-induced craving: VAS score  Anxiety: SAS  Depression: SDS  Withdrawal symptoms: withdrawal symptom scale for MA addicts | iTBS and 10 Hz rTMS resulted in similar reductions in craving, and both improved withdrawal symptoms. rTMS significantly improved affective symptoms.  No significant differences in outcomes between iTBS vs rTMS. |
| Zhao et al., 2020 (61) | 83  27 iTBS left  26 cTBS left  30 cTBS right  MUD | iTBS over left DLPFC  Vs  cTBS over right DLPFC  *Twice daily for 10 total sessions* | cTBS over left DLPFC  *Twice daily for 10 total sessions* | Cue-induced craving: VAS score  Sleep quality: PSQI  Depression: BDI  Anxiety: BAI  Impulsivity: BIS-11  Adverse effects: self-report | iTBS of the left DLPFC and cTBS of the right DLPFC showed a reduction in craving, although cTBS over the right DLPFC did not.  cTBS L and iTBS significantly improved sleep. Only iTBS improved anxiety.  Significant improvement in depressive symptoms across all groups.  Adverse effects were higher in the left iTBS group. |
| Sanna et al., 2019 (62) | 47  25 iTBS  22 rTMS  CUD | Bilateral iTBS over PFC | 15 Hz rTMS over PFC | Craving: CCQ, VAS  Complication from drug use likelihood: WHO ASSIST  Cocaine consumption: urine test and self-reporting | Virtually identical significant effects on outcomes observed between iTBS and rTMS, with similar side effects and dropout rates.  Both iTBS and rTMS significantly reduced cocaine intake, with no significant difference between the two. |
| Review studies | | | | | |
| Author, Year  Type of review | Sample size (n) | Number of studies in review | Types of studies | Focus of review | Findings/Implications/gaps/future |
| Wu et al., 2022 (6)  Network meta-analysis | 1888 | 22 | RCTs | Compare efficacy and acceptability of various NIBS methods/protocols for AUD/MUD management | Largest decreases in craving were found when a combination of iTBS over left DLPFC and cTBS over left vmPFC were used.  High frequency rTMS over left DLPFC was associated with the largest improvements in depression and sleep quality.  Need larger trials focusing on cTBS. |
| Moretti et al., 2020 (95)  Literature review | Not stated | Not stated | Not stated | rTMS-induced changes in glutamatergic and dopaminergic systems in the treatment of CUD and MUD.  Discussed evidence on types of NIBS: rTMS, HF vs LF, iTBS, cTBS. | Need studies with longer follow-up and better consistency across stimulation parameters.  Expand outcomes of interest beyond subjective craving. |
| Alba-Ferrara et al., 2014 (96)  Literature review | Not stated | Not stated | Not stated | Neuromodulation in CUD | Pharmacological treatments have had limited efficacy. Not all patients respond to behavioural treatments.  To prevent needing invasive DBS, whilst still achieving similar efficacy, dTMS was created.  Future studies should combine multiple measures to assess drug use (urine tests, self-reports and questionnaires).  More studies using fMRI and EEG to understand mechanisms. |

Diagnosis abbreviations: CUD (Cocaine Use Disorder), MUD (Methamphetamine Use Disorder), AUD (Alcohol Use Disorder)

Brain region abbreviations: DLPFC (dorsolateral Prefrontal Cortex), PFC (Prefrontal Cortex), vmPFC (Ventromedial Prefrontal Cortex)

Technology abbreviations: rTMS (Repetitive Transcranial Magnetic Stimulation), iTBS (intermittent Theta-Burst Stimulation), cTBS (Continuous Theta-Burst Stimulation), HF (High Frequency), LF (Low Frequency), NIBS (Non-Invasive Brain Stimulation), dTMS (Deep Transcranial Magnetic Stimulation), DBS (Deep Brain Stimulation), fMRI (functional Magnetic Resonance Imaging), EEG (Electroencephalogram)

Outcome assessment abbreviations: VAS (Visual Analogue Scale), SAS (Self-rating Anxiety Scale), SDS (Zung Self-rating Depression Scale), PSQI (Pittsburgh Sleep Quality Index), BDI (Beck Depression Inventory), BAI (Beck Anxiety Inventory), BIS-11 (Barratt Impulsiveness Scale), CCQ (Cocaine Craving Questionnaire), WHO ASSIST (World Health Organization Alcohol Smoking and Substance Involvement Screening Test)

Study design: RCT (Randomized Controlled Trial)
