# Supplementary material for: Non-invasive neurostimulation techniques for the treatment of stimulant use disorders
Source: Front Psychiatry. 2026 Feb 4;17:1755441. doi: 10.3389/fpsyt.2026.1755441 (PMC12913103; doi:10.3389/fpsyt.2026.1755441)
Supplement: Supplementary file 3 [file Table3.docx]

Supplementary Material

**Supplementary Table 3 – Extended results of all included studies with a focus on comparing target sites**

| Author, Year | Sample size (n)  Participant diagnosis | Target site | Intervention | Comparator | Outcome of interest | Findings |
| --- | --- | --- | --- | --- | --- | --- |
| Non-review studies | | | | | | |
| Hou et al., 2025 (37) | 60  20 Active left DLPFC  20 Active right DLPFC  20 Sham  MUD | Right vs Left  DLPFC | 8 consecutive days of HD-tDCS at 1.5mA to either left DLPFC or right DLPFC | 1.5 mA (first 30 seconds), 0 for remainder sham HD-tDCS | Drug craving as measured by: Physiological: heart rate and blood pressure  Explicit: VAS score  Implicit: SC-IAT | Intervention group: showed significant decreases in heart rate and drug craving scores between pre- and post-treatment. No difference between left and right.  Sham group: no significant differences pre- and post-treatment.  1 month follow-up:  Explicit craving scores in the intervention group significantly lower than pre-test scores, although significantly higher than post-test scores. |
| Rezvanian et al., 2022 (16) | 15 male  MUD | Right DLPFC anodal + left DLPFC cathodal  Vs  Right DLPFC cathodal + left DLPFC anodal  Vs  Right DLPFC anodal + Right arm cathodal  Vs  Left DLPFC anodal + Left arm cathodal  Vs  Right cerebellar hemisphere (O2) anodal + Left cerebellar hemisphere (O1) cathodal  Vs  Right cerebellar hemisphere (O2) cathodal + Left cerebellar hemisphere (O1) anodal | 2 mA tDCS - single session of each protocol with a 72-hour washout period between them | Each protocol vs the other | Craving: VAS score  Inhibition: Go/no-go task  Working memory: N-back task | Craving was not significantly changed by tDCS.  Significant increase in cognitive inhibition, with the greatest effect being seen in protocol 2.  Significant improvement for presentation error was seen with tDCS, with the highest effect being seen in protocol 4. |
| Chen et al., 2020 (38) | 74  A: 18  B: 18  C: 19  D (sham): 19  MUD | 10 sessions across 2 weeks (1 per day, 5 days per week). 3-pulse 50 Hz bursts  Group A: Left DLPFC  vs  Group B: Left vmPFC  Vs  Group C: A+B | Group A: iTBS  Group B: cTBS | Sham TBS | Cue-induced craving: VAS score  Anxiety: HAMA-14  Depression: HAMD  Sleep quality: PSQI  Cognition: various scales and tests  Withdrawal symptoms: AWQ | Active TBS significantly reduced craving compared to sham regardless of site.  Group C significantly outperformed Group A on anxiety and withdrawal symptoms. Non-significant improvements were seen in depression and sleep quality.  No significant changes to cognition. |
| Shahbabaie et al., 2018 (68) | 90  16 in each montage  16 in sham  MUD | 5 different electrode montages:  Anodal tDCS over right DLPFC with return electrode over left shoulder  Vs  Anodal tDCS over right DLPFC with return electrode over left supraorbital ridge  Vs  Anodal over Left DLPFC with return electrode over right shoulder  Vs  Anodal over Left DLPFC with return electrode over right supraorbital ridge  Vs  Anodal over Left DLPFC with return electrode over contralateral DLPFC | 2 mA tDCS | Sham tDCS (right DLPFC and left DLPFC) | Attentional Bias: Probe detection task  Depression + Anxiety: PANAS | Left DLPFC/right shoulder and left DLPFC/rightDLPFC reduced attentional bias towards drug cues compared to sham.  No significant differences in affective symptoms between groups. |
| Liu et al., 2017 (58) | 50  10 in each group  MUD | Left P3  Left DLPFC  Right DLPFC | rTMS - low and high frequency:  10 Hz Left P3  10 Hz Left DLPFC  10 Hz Right DLPFC  1 Hz Left DLPFC  1 Hz Right DLPFC | Site vs site, low vs high frequency | Cue-induced craving: score from 0-100 following playing with tools of drug use and fake methamphetamine for 5 minutes | Right or left side rTMS effectively reduced cue-induced craving in both low and high frequency settings.  Active rTMS stimulation at P3 point did not reduce craving. |
| Camprodon et al., 2007 (59) | 6  CUD | Right vs left DLPFC | 2 sessions of 10 Hz rTMS over left or right DLPFC | N/A | Craving: VAS score  Anxiety: self report  Mood: self-report: Happiness vs sadness | Right, but not left, DLPFC stimulation resulted in a significantly reduced craving, however this disappeared after 4 hours.  Significant improvement in anxiety and mood symptoms. |
| Review studies | | | | | | |
| Author, Year  Type of review | Sample size (n)  Participant diagnosis | Number of studies in review | Types of studies | Focus of review | Findings | Implications/gaps/future |
| Edinoff et al., 2023 (97)  Literature review | Not available | Not available | Not available | Transcranial stimulation for stimulant use disorder | TMS reduces risk factors associated with relapse | Most studies focus on males  More generalizable study populations are required  More neuroimaging studies |
| Liu et al., 2021 (98)  Literature review | 828 | 34 | RCT, 1 cross-over | NIBS target  in substance use disorders  Scope included tobacco, alcohol, opioids and stimulants | All studies across each disorder focused on DLPFC which was found to be an effective target for all addictive substances.  Side effects reported across stimulant studies included mild scalp discomfort, dizziness  and fatigue. | Further research required to validate DLPFC as primary target  Combine NIBS with behavioural therapy |
| Protasio et al., 2019 (99)  Literature review | 91 | 7 | Mostly RCTs | rTMS for reducing cocaine craving and use | Studies focused on either DLPFC or mpFC, both good targets clinically..  Right vs left DLPFC was also assessed across studies. Only 1 trial found R>L, other found L or bilateral to be superior | Need larger study samples and more studies which include a control. |

Diagnosis abbreviations: CUD (Cocaine Use Disorder), MUD (Methamphetamine Use Disorder)

Brain region abbreviations: DLPFC (dorsolateral Prefrontal Cortex), MPFC (Medial Pre-frontal Cortex), vmPFC (Ventromedial Prefrontal Cortex)

Technology abbreviations: rTMS (Repetitive Transcranial Magnetic Stimulation), tDCS (Transcranial Direct Current Stimulation), HD-tDCS (High-Definition Transcranial Direct Current Stimulation), iTBS (intermittent Theta-Burst Stimulation), cTBS (continuous Theta-Burst Stimulation), TMS (Transcranial Magnetic Stimulation), NIBS (Non-Invasive Brain Stimulation)

Outcome assessment abbreviations: VAS (Visual Analogue Scale), SC-IAT (Single Category Implicit Association Test), HAMA-14 (Hamilton Anxiety Rating Scale, 14 item), HAMD (Hamilton Rating Scale for Depression), PSQI (Pittsburgh Sleep Quality Index), AWQ (Amphetamine Withdrawal Questionnaire), PANAS (Positive and Negative Affect Score)

Study design: RCT (Randomized Controlled Trial)
