# Supplementary material for: Non-invasive neurostimulation techniques for the treatment of stimulant use disorders
Source: Front Psychiatry. 2026 Feb 4;17:1755441. doi: 10.3389/fpsyt.2026.1755441 (PMC12913103; doi:10.3389/fpsyt.2026.1755441)
Supplement: Supplementary file 4 [file Table4.docx]

Supplementary Material

**Supplementary Table 4 – Extended results of all included studies with a focus on neuroimaging outcomes**

| Author, Year | Sample size (n)  Participant diagnosis | Intervention | Comparator | Neuroimaging modality | Outcome of interest | Findings |
| --- | --- | --- | --- | --- | --- | --- |
| Non-review studies | | | | | | |
| Zhang et al., 2025 (63) | Discovery cohort:  41 CUD  44 controls  Independent cohort:  53 CUD  45 controls  Treatment cohort:  44 CUD | 10 sessions of 5 Hz rTMS to left DLPFC over 2 weeks | Healthy controls only used for their fMRI findings | fMRI | Craving: VAS score  fMRI changes post rTMS vs baseline: fMRI | At baseline across all 3 cohorts, CUD patients showed elevated gradient values in the ventral striatum.  rTMS significantly normalised gradient values in the ventral striatum and this was seen to be in correlation with decreases in craving. This association was significant. |
| Li et al., 2024 (45) | 34  17 Active rTMS  17 Sham  MUD  17 Healthy controls | Twice weekly sessions of 10 Hz rTMS over DLPFC to a total of 8 sessions | Sham rTMS in MUD  Healthy controls | EEG | Changes in microstates: EEG  Craving: VAS score | 2 of the 4 microstates (which were lower in duration in MUD patients at baseline) showed significant improvements after rTMS and these were found to be significantly correlated with reductions in craving level. |
| McCalley et al., 2024 (20) | 33  17 cTBS  16 sham  CUD | 10 sessions of continuous theta burst stimulation (cTBS) over left frontal pole of the MPFC across 3 weeks in combination with behavioural counselling | Sham cTBS + behavioural counselling | fMRI | Brain activity in response to cues  Craving | Reduced activity in MPFC, insula and anterior cingulate.  No change to craving. |
| Rasgado-Toledo et al., 2024 (39) | 50  27 Active rTMS  23 Sham  CUD | 2 daily sessions of rTMS on DLPFC, for a total of 20 sessions | Sham rTMS | T1-weighted and high angular resolution diffusion-weighted imaging. | Craving: VAS score and CCQ-now  MRI changes to white matter (WM) microstructure in the frontostriatal circuits. | Active rTMS resulted in a significant increase in neurite density compared to sham in WM tracts connecting left DLPFC with left and right vmPFC.  rTMS also showed reduction in orientation dispersion in WM tracts connecting the left DLPFC with the left caudate nucleus, left thalamus and left vmPFC.  None of these changes correlated to changes in craving or impulsivity.  A greater reduction in craving was noted in patients who had a low baseline ICVF in WM tracts connecting the left caudate nucleus with substantial nigra. |
| Chen et al., 2023 (69) | 19  10 Active tDCS  9 Sham  MUD (amphetamine)  11 Healthy controls | tDCS daily for 5 days with cathode over left DLPFC and anode over right DLPFC | Sham tDCS  Healthy controls only used for baseline EEG/ERP | EEG  ERP Scalp topographies | Behavioural performance with concurrent EEG: Posner cueing task  P300 amplitudes | tDCS resulted in an increased amplitude of P300 to neutral cues, to a significant level for neutral cues, trending towards the healthy controls. P300 amplitude insignificantly increased to drug-related cues.  No significant effect was noted on behavioural performance, but an effect nearing significance was seen in the tDCS group.  Changes in disengagement scores showed a significant reduction in the tDCS group. |
| Zhao et al., 2023 (100) | Discovery cohort  71 CUD  58 healthy controls  Independent cohort  81 CUD  82 healthy controls  Experimental (to receive active or sham rTMS)  45 CUD | 5 Hz rTMS to left DLPFC | Sham rTMS | fMRI | Treatment response with diagnostic FC signature | Applying the functional connectivity signature to rTMS therapy was found to be predictive for treatment response |
| Ekhtiari et al., 2022 (40) | 60 male  30 Active tDCS  30 Sham  MUD | Active 2 mA tDCS over F4/Fp1 - single session | Sham tDCS | fMRI | Craving: VAS Score  Brain activity changes: fMRI | No significant changes to cravings were observed after the single session.  A decrease in brain activity in response to drug cues during fMRI after sham tDCS was observed. Whilst active tDCS participants showed a significant  increase in brain activity in response to drug cues and stronger connections within the frontoparietal network were observed. |
| Khajehpour et al., 2022 (17) | 42 (male)  22 Active tDCS  22 Sham (2 excluded due to poor EEG data)  MUD | Single session 2 mA tDCS over DLPFC | Sham tDCS | EEG | Cue-induced craving: VAS score  Biased attention to drug stimuli  P3 and Late Positive Potential (LPP): EEG | P3 amplitude (initial attentional bias) significantly decreased in the active tDCS group in response to drug-related cues, whilst it increased in the sham group.  No significant change to LPP (sustained motivated attention) was noted in response to drug related cues following tDCS.  Significant change to craving. |
| Soleimani et al., 2022 (15) | 15  MUD | 2 mA tDCS over F4/F3 - single session | Sham tDCS - same participants after washout period | MRI - both structural and functional during a drug cue-reactivity task | MRI changes to 3 identified relevant brain areas:   - Executive control network (ECN) - Default Mode Network (DMN) - Ventral Attention Network (VAN)   Craving: VAS score | Active rTMS increased the activity and communication between the ECN and VAN, whilst it decreased the activity and communication between the DMN and VAN.  Cravings decreased in the active rTMS group. |
| Wen et al., 2022 (46) | 15 (all female)  MUD  *Distribution between active and control not stated.* | iTBS | Sham iTBS | EEG | Craving: VAS score  Theta:Beta ratio: EEG | Significant reduction of craving was noted in the active iTBS group.  Theta:beta ratio was decreased significantly in active iTBS group |
| Chen et al., 2021 (47) | 49  35 Active iTBS  22 Sham  MUD | 20 sessions of iTBS over DLPFC | Sham iTBS | EEG | Addiction Stroop Task whilst EEG monitoring  Craving: VAS | Reduced error rate was observed in the active group compared to sham.  Active group also showed stronger P3 amplitudes (connected to a faster response on task) although not significant. Reduction in beta-wave activity in the frontal lobe of the brain compared to the sham group.  Cravings correlated to changes in N1 amplitude on EEG. |
| Garza-Villarreal et al., 2021 (25) | 44 - initial 2 week phase  CUD  20 - continued onto 6/12 maintenance phase  CUD | 2 daily sessions of rTMS on left DLPFC PLUS standard treatment for 2 weeks  Maintenance: 2 weekly sessions for up to 6 months | N/A | fMRI | Brain changes: fMRI  Craving: VAS | Increased connectivity between the left DLPFC and vmPFC, and between the vmPFC and right angular gyrus were observed following rTMS. These effects remained until 3 months, and were gone by 6 months.  Craving was reduced over the first 2 weeks. |
| Su et al., 2020 (42) | 50  25 Active rTMS  25 Sham  MUD | 4-week course 5 Hz rTMS over left DLPFC to total of 20 sessions | Sham rTMS | Proton magnetic resonance spectroscopy (H MRS) | GABA levels and  Resonance of glutamate and glutamine (Glx) in left DLPFC relative to n-acetyl-aspartate (NAA): H MRS  Correlate these with clinical outcomes such as craving and cognitive function: VAS + CogState battery | Significant reductions in GABA/NAA were observed in the active rTMS group. Sham did not show this.  Significant reduction in Glx and NAA in sham rTMS not active.  Significant association between reductions in GABA and improvement in cognitive function.  Cravings were significantly decreased in active but not in sham. |
| Su et al., 2020 (41) | 60  30 Active iTBS  30 Sham  MUD | 20 sessions of iTBS  (short bursts of 50 Hz rTMS repeated at a rate in the theta range (5 Hz), 2-sec on, 8-sec off  for 5 min; 900 pulses) over DLPFC | Sham rTMS | fMRI | Craving: VAS score  Connectivity of brain areas: fMRI | Increased connectivity was observed between the left DLPFC and inferior parietal lobule in active iTBS and this was correlated to a reduction in craving.  Decreased connectivity between insula and inferior parietal lobule, medial temporal lobe and precuneus was also observed in those in active iTBS. |
| Kearney-Ramos et al., 2019 (43) | 19  CUD | cTBS to mPFC | Sham cTBS (same participants - cross over study) | fMRI | Brain changes during cue exposure: fMRI  Craving: self-reported 1-10 | In active cTBS, the striatum became less active following the treatment when exposed to cues. The extent of this change was correlated to how active the striatum was before treatment, with those showing a greater baseline response to drug cues, experiencing a greater reduction post treatment.  No significant change to craving. |
| Shahbabaie et al., 2018 (44) | 15 (male)  MUD | 2 mA tDCS over DLPFC | Sham tDCS (same participants - crossover  after 1 week washout) | fMRI | Craving: subjective rating from 0-100  fMRI focused on ECN, DMN and salience network (SN) | Craving decreased significantly in active tDCS compared to sham and this was correlated with the changes observed in brain networks with significant modulation of DMN, ECN and SN noted after active tDCS compared to sham. |
| Nakamura-Palacios et al., 2016 (64) | 14  CUD | 2 mA tDCS cathodal left DLPFC and anodal right DLPFC, five days in a row | N/A | EEG - ERPs  MRI - DTI | Cue-induced craving  Brain changes: ERPs and DTI | Brain connections between vmPFC and nucleus accumbens became stronger after real tDCS and these stronger connections were related to a reduction in craving |
| Conti et al., 2014 (101) | 13  7 Active tDCS  6 Sham  CUD | 1/day, every other day, bilateral 2 mA tDCS for total of 5 sessions over DLPFC | Sham tDCS | EEG | Brain activity during neutral or crack-related cues: EEG | After a single session of active tDCS, P3 current density increased in the left DLPFC during neutral cues and decreased during crack-related cues. The opposite was seen in sham.  Following repeated tDCS sessions, P3 was increased in left DLPFC as well as other areas including FPC, OFC and ACC in response to crack-related cues in the active group. |
| Review studies | | | | | | |
| Author, Year  Type of review | Sample size (n)  Participant diagnosis | Number of studies in review | Types of studies | Focus of review | Findings | Implications/gaps/suggestions |
| Shen et al., 2021 (102)  Literature review | 289 | 6 | Primary studies in humans | TMS with neuroimaging for CUD | Drug cue reactivity decrease  Informative on who will likely respond (higher values of fractional anisotropy)  No serious adverse events reported in any of the studies  People with SUDs have altered functional connectivity and cortical excitability. | Proposed combining TMS with neuroimaging will accelerate understanding.  Network biomarkers of substance use need to be identified so we can manipulate them to improve therapy. |

Diagnosis abbreviations: CUD (Cocaine Use Disorder), MUD (Methamphetamine Use Disorder), SUD (Substance Use Disorder)

Brain region abbreviations: DLPFC (dorsolateral Prefrontal Cortex), MPFC (Medial Pre-frontal Cortex), vmPFC (Ventromedial Prefrontal Cortex), WM (White Matter), ECN (Executive Control Network), DMN (Default Mode Network), VAN (Ventral Attention Network), SN (Salience Network), FPC (Frontopolar Cortex), OFC (Orbitofrontal Cortex), ACC (Anterior Cingulate Cortex)

Technology abbreviations: rTMS (Repetitive Transcranial Magnetic Stimulation), tDCS (Transcranial Direct Current Stimulation), iTBS (intermittent Theta-Burst Stimulation), cTBS (Continuous Theta-Burst Stimulation), TMS (Transcranial Magnetic Stimulation), fMRI (functional Magnetic Resonance Imaging), EEG (Electroencephalogram), ERP (Event Related Potential), FC (Functional Connectivity), LPP (Late Positive Potential), DTI (Diffusor Tensor Imaging)

Outcome assessment abbreviations: VAS (Visual Analogue Scale), CCQ (Cocaine Craving Questionnaire), ICVF (Intra-cellular Volume Fraction)
